# Global Aromaticity and Antiaromaticity in Porphyrin Nanoring Anions†

**DOI:** 10.1002/anie.201909032

**Published:** 2019-09-17

**Authors:** Martin D. Peeks, Michael Jirasek, Timothy D. W. Claridge, Harry L. Anderson

**Affiliations:** ^1^ Department of Chemistry University of Oxford Chemistry Research Laboratory Oxford OX1 3TA UK; ^2^ School of Chemistry University of New South Wales Sydney NSW 2052 Australia

**Keywords:** anions, annulenes, aromaticity, macrocycles, porphyrinoids

## Abstract

Doping, through oxidation or reduction, is often used to modify the properties of π‐conjugated oligomers. In most cases, the resulting charge distribution is difficult to determine. If the oligomer is cyclic and doping establishes global aromaticity or antiaromaticity, then it is certain that the charge is fully delocalized over the entire perimeter of the ring. Herein we show that reduction of a six‐porphyrin nanoring using decamethylcobaltocene results in global aromaticity (in the 6− state; [90 π]) and antiaromaticity (in the 4− state; [88 π]), consistent with the Hückel rules. Aromaticity is assigned by NMR spectroscopy and density‐functional theory calculations.

## Graphical abstract

**Negative isn't always bad**: Reduction of a porphyrin nanoring by the addition of 4 or 6 electrons was found to result in global (anti)aromaticity (see picture), as revealed by ^1^H NMR spectroscopy and DFT calculations. Thus, the Hückel rules apply to huge macrocyclic anions, and the charge in these anions is fully delocalized.

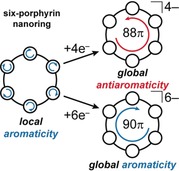

Aromaticity is one of the oldest concepts in chemistry, and it is widely used to predict the properties of unsaturated cyclic molecules.^1^ In essence, aromaticity describes the anomalous stability of certain molecules, and their peculiarly large anisotropic diamagnetic susceptibilities.^2^ This unusual diamagnetism results from the tendency of aromatic molecules to sustain a ring current when placed in a magnetic field. This effect is easily detected by NMR spectroscopy, but aromatic ring currents were first postulated by Pauling and Lonsdale before NMR spectroscopy had been invented.^3^ While the earliest aromatic molecules were flat symmetric carbocyclic π‐systems, the concept has been extended to σ‐bonded systems, metal clusters, excited states, twisted molecules, and other systems.^4^, ^5^, ^6^

Hückel's calculations predict that an annulene (C_*n*_H_*n*_) with a conjugated circuit of [4*n*+2] π‐electrons will be aromatic.^7^ Breslow extended this rule to state that annulenes with [4*n*] π‐electrons are antiaromatic, with reduced stability and the opposite ring currents (paratropic rather than diatropic).^8^ Although Hückel's original calculations were restricted to all‐carbon, monocyclic annulenes, the rules also apply to a wide range of heterocyclic and polycyclic molecules. However, there are many macrocyclic π‐conjugated rings with [4*n*+2] or [4*n*] π‐electrons that do not display global aromatic or antiaromatic ring currents. For example, cycloparaphenylenes ([*n*]CPPs, Figure 1) have [4*n*] π‐electrons in their circumferential conjugation path, yet are non‐aromatic in their electronically neutral ground states.^9^ Similarly, butadiyne‐linked porphyrin nanorings, such as ***c*** **‐P6⋅T6**, do not exhibit global (anti)aromaticity in their neutral states.^10^, ^11^, ^12^ These macrocycles consist of aromatic subunits, and the local aromaticity of the subunit dominates in the neutral ground states. (Anti)aromaticity can be induced by perturbing the electronic structure, for example, by oxidation or excitation. The removal of two electrons from [8]CPP leads to global aromaticity (30 π‐electrons).^9^ Removal of 4 or 6 electrons from the six‐porphyrin nanoring ***c*** **‐P6⋅T6** leads to macrocyclic global antiaromaticity (80 π‐electrons) or aromaticity (78 π‐electrons), respectively.^13^ Electronic excitation can lead to excited‐state aromaticity,^4^, ^14^ and molecules such as ***c*** **‐P6** are aromatic in their lowest excited states.^15^ Many other macrocycles with global aromaticity in the neutral, cationic, and excited states have been reported in the last decade.^16^, ^17^, ^18^, ^19^, ^20^, ^21^

**Figure 1:**
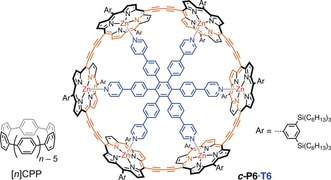
Examples of π‐conjugated macrocycles: [*n*]cycloparaphenylenes (*[n]*CPPs), and a six‐porphyrin nanoring, ***c*** **‐P6⋅T6** (template **T6** in blue; conjugated circuit in orange).

Porphyrin oligomers have an exceptionally wide range of accessible oxidation states; for example, ***c*** **‐P6⋅T6** can be reversibly oxidized up to the 12+ state and reduced to the 6− state.^13^, ^22^ Herein we demonstrate that global ring currents can also be established by reduction. We show that the tetraanion is antiaromatic (88 π‐electrons), and the hexaanion is aromatic (90 π‐electrons). The chemical reduction of annulenes was widely explored during the 1980s,^23^ but there are few recent examples of macrocycles that become aromatic in anionic reduced states,^19^ and it was not clear whether this strategy could be used in complex heterocyclic macrocycles, such as porphyrin nanorings.

We began this study by using computational techniques to predict the (anti)aromaticity in reduced states of ***c*** **‐P6⋅T6**. The nucleus‐independent chemical shift (NICS),^24^ which evaluates the chemical shift at a point in space, was calculated using density functional theory (DFT, B3LYP/6‐31+G*) for the template‐free ***c*** **‐P6**. The diatropic ring currents of aromatic molecules generate an induced field that opposes the external magnetic field inside the ring, thus leading to a shielding effect and lower chemical shifts. Outside the ring, the induced field reinforces the applied field, leading to NMR deshielding. The paratropic ring currents of antiaromatic molecules have the opposite NMR effects. Therefore, a negative NICS inside the nanoring indicates aromaticity, and a positive NICS indicates antiaromaticity.

The results of NICS calculations (Figure 2 and Table 1) are consistent with predictions from the Hückel rule: the 4− state is antiaromatic (NICS(0)_iso_=89 ppm), and the 6− state is aromatic (NICS(0)_iso_=−13 ppm). These NICS values are similar to those calculated previously for the 4+ and 6+ oxidation states.^13^ The prediction of aromaticity in the 6− state is consistent across other functionals (M06‐2X and ωB97XD; see Table S1 in the Supporting Information). The 4− state undergoes symmetry breaking from *D*_6*h*_ to approximate *C*_2*v*_ symmetry, reflecting the pseudo‐Jahn–Teller distortion common to antiaromatic molecules. The functionals M06‐2X and ωB97XD predict greater ellipticity than B3LYP (see Table S2), and corresponding lower NICS values (see Table S1). Calculations of the anisotropy of the induced current density (ACID) confirm the conclusions from the NICS calculations (see Figure S3).^25^

**Figure 2:**
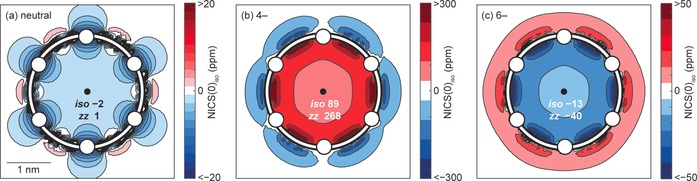
NICS(0)_iso_ grids for ***c*** **‐P6** at the B3LYP/6‐31+G* level of theory: a) neutral; b) 4− state; c) 6− state. The location of porphyrin subunits is indicated by white circles. The NICS(0)_iso_ and NICS(0)_*zz*_ values at the centers of the rings are indicated at the center of each plot. Further NICS grids and results from other levels of theory are available in the Supporting Information (see Table S1 and Figures S1 and S2).

**Table 1:** Nucleus‐independent chemical shifts for ***c*** **‐P6** as a function of oxidation state, at B3LYP/6‐31+G*.

	Neutral	4−	6−	4+^[a]^	6+^[a]^
*n* π e^−^	84	88	90	80	78
NICS(0)_iso_	−1.5	88.7	−13	101	−13

[a] Data from ref. 13.

Square‐wave voltammetry of ***c*** **‐P6⋅T6** revealed six reversible reductions in the window −1.30 to −1.80 V (vs. Fc/Fc^+^).^22^ There are few reducing agents available in this potential range.^26^ Alkali metals are strong reducing agents, but it is difficult to titrate them to avoid over‐ or under‐reduction. Therien and co‐workers used decamethylcobaltocene (CoCp*_2_) to prepare radical anions of linear porphyrin oligomers.^27^ CoCp*_2_ is a convenient reducing agent because it is sufficiently soluble in THF, and it has an oxidation potential of −1.84 V vs. Fc/Fc^+^,^26^ making it strong enough to access the 6− oxidation state of ***c*** **‐P6⋅T6**.

Addition of excess CoCp*_2_ to a solution of ***c*** **‐P6⋅T6** in [D_8_]THF resulted in a red‐brown solution with a fairly well resolved ^1^H NMR spectrum corresponding to ***c*** **‐P6⋅T6^6−^**. Addition of [D_5_]pyridine (5 % by volume of solvent) resulted in a slightly sharper spectrum (Figure 3 c). Reduction was reversible and the addition of ferrocenium hexafluorophosphate cleanly regenerated ***c*** **‐P6⋅T6** (see Figure S7). Addition of 4 equivalents of CoCp*_2_ to neutral ***c*** **‐P6⋅T6** gave the 4− state, characterized by a broad chemical‐shift dispersion (signals up to 18 ppm, Figure 3 b). Before assigning the ^1^H NMR spectra of ***c*** **‐P6⋅T6^4−^** and ***c*** **‐P6⋅T6^6−^**, it is helpful to recapitulate the assignment of neutral ***c*** **‐P6⋅T6** (Figure 3 a). The protons of the solubilizing trihexylsilyl groups (THS_i_ and THS_o_ for those pointing inside and outside the ring, respectively) give rise to a broad overlapping set of peaks around 1 ppm. The porphyrin β‐pyrrole hydrogen atoms (labeled a and b in Figure 3) resonate at *δ*=9.6 and 8.8 ppm, respectively, and the proton resonances of the *meso*‐aryl groups (labeled *o*_o_, *o*_i_, and *p*) appear in the expected region for aromatic signals (*δ*=8.1–8.4 ppm). In contrast, the template protons (α, β, γ, and δ) are substantially (up to 6 ppm) shielded by their axial coordination to the porphyrin subunits, which are each locally aromatic.

**Figure 3:**
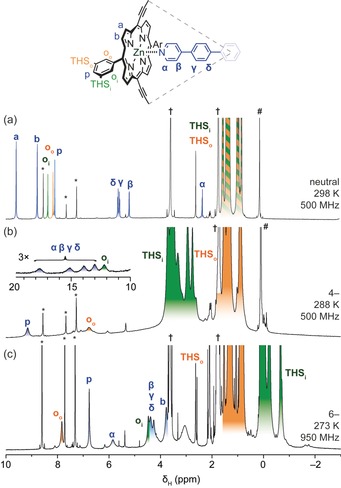
^1^H NMR spectra of ***c*** **‐P6⋅T6** in its a) neutral, b) 4−, and c) 6− oxidation states. (THS=trihexylsilyl. Known impurities and solvent signals are labeled: * pyridine; † THF; # silicone grease. Full‐scale spectra are shown in Figure S6 of the Supporting Information. We did not observe any field dependence in the chemical shifts other than slight shifts associated with chemical exchange processes.)

The global ring current around the nanoring can be evaluated by comparing the shielding inside and outside the nanoring (i.e. THS_i_ vs. THS_o_ and *o*_i_ vs. *o*_o_). The resonances of the template protons (α, β, γ, and δ) in ***c*** **‐P6⋅T6^6−^** are readily identified at approximately 4–5 ppm by comparison with the spectrum of template‐free ***c*** **‐P6^6−^** (see Figure S8). The resonances of the *meso*‐aryl protons (*o*_i_, *o*_o_, and *p*) are each related by a ^4^ *J* scalar coupling, and so exhibit a characteristic coupling pattern in the ^1^H TOCSY spectrum (see Figure S9). NOE correlations between the template protons and *o*_i_ permit the assignment of the latter, and further NOE correlations to the THS manifold centered around −0.5 ppm confirm its assignment as THS_i_ (see Figure S11). THS_o_ and *o*_o_ are related by NOE correlations, and *p* can then be assigned by elimination. Although the THS_i_ resonance is shielded by the global aromatic ring current, the THS_o_ resonance is at the same chemical shift as for the neutral ring because these protons sit at the border between the shielding and deshielding regions of the chemical‐shift‐anisotropy cone (see Figure S2). The chemical‐shift difference between resonances on the inside and outside of the nanoring, Δ*δ*, gives a measure of global (anti)aromaticity. For ***c*** **‐P6⋅T6^6−^**, Δ*δ*_o_=−3.35 ppm, and Δ*δ*_THS,CH3_=−1.18 ppm. These values are approximately 1.75 times higher than those for the aromatic hexacation, ***c*** **‐P6⋅T6^6+^**,^13^ thus suggesting that the hexaanion has a stronger global diatropic ring current than the hexacation.

The spectrum of the 4− oxidation state is much broader than that of the 6− state, which made it impossible to record useful 2D ^1^H TOCSY and ^1^H–^13^C HSQC spectra of ***c*** **‐P6⋅T6^4−^**, but NOESY spectra provided valuable correlations. The THS_o_ resonance is readily assigned, since it is unperturbed by global (anti)aromaticity. It can then be seen that the THS_i_ resonance is deshielded (appearing at ca. 3–4 ppm), consistent with global antiaromaticity. NOEs between THS_i_ and *o*_i_, and between THS_o_ and *o*_o_, permit the assignment of the signals for the *ortho* hydrogen atoms (see Figure S17). Both THS resonances also have equal‐intensity NOEs to the *p* resonance. The α–δ template resonances at 13–17 ppm were identified by comparing the spectra of ***c*** **‐P6⋅T6^4−^** and template‐free ***c*** **‐P6^4−^** (Figure 3 b; see also Figure S16); the extreme deshielding of these protons supports the assignment of antiaromaticity.

The energy barrier for rotating one porphyrin subunit by 180° in the template‐free ***c*** **‐P6** nanoring (swapping THS_i_ and THS_o_, *o*_i_ and *o*_o_) is very sensitive to the oxidation state. The transition state for this process must break the π‐conjugation, so the barrier provides a probe for the enhanced resonance energy in charged species. In neutral ***c*** **‐P6**, rotation of porphyrin units is fast at room temperature. Upon cooling, the *o*_i_/*o*_o_ resonances separate and enter the slow exchange regime, with a coalescence temperature *T*_c_ of 200–203 K, corresponding to a barrier of (38.0±0.5) kJ mol^−1^ (see Figure S22). EXSY experiments on ***c*** **‐P6^6−^** at 228 K revealed a rotation barrier of (50.2±0.5) kJ mol^−1^ (see Figure S25 and Table S4), which is similar to that previously measured for ***c*** **‐P6^6+^** ((49.5±0.4) kJ mol^−1^ at 213 K).

In conclusion, we have shown that [90 π] aromatic and [88 π] antiaromatic anions can be generated by chemical reduction of a six‐porphyrin nanoring of diameter 2.5 nm using decamethylcobaltocene. This approach to establishing global (anti)aromaticity is probably applicable to many other redox‐active π‐conjugated macrocycles and provides a useful complement to chemical oxidation. Since larger aromatic‐ring‐current effects are observed by NMR spectroscopy for ***c*** **‐P6⋅T6^6−^** than for ***c*** **‐P6⋅T6^6+^**, reduction may prove more useful than oxidation for establishing global aromaticity in even larger macrocycles. Aromatic macrocycles with diameters on the order of 10–20 nm are expected to show mesoscale magnetic properties, such as a nonlinear dependence of the induced ring current on the applied magnetic field, similar to that observed in mesoscopic metal rings.^28^

Cartesian coordinates for computational structures are available in the Supporting Information and from https://doi.org/10.6084/m9.figshare.9405308.

## Conflict of interest

The authors declare no conflict of interest.

## Supporting information

As a service to our authors and readers, this journal provides supporting information supplied by the authors. Such materials are peer reviewed and may be re‐organized for online delivery, but are not copy‐edited or typeset. Technical support issues arising from supporting information (other than missing files) should be addressed to the authors.

Supplementary
