# Supplementary material for: Global Aromaticity and Antiaromaticity in Porphyrin Nanoring Anions
Source: Angew Chem Int Ed Engl. 2019 Sep 17;58(44):15717–20. doi: 10.1002/anie.201909032 (PMC6856819; doi:10.1002/anie.201909032)
Supplement: Supplementary file 1 — Supplementary [file ANIE-58-15717-s001.pdf]

Angewandte Chemie
Eine Zeitschrift der Gesellschaft Deutscher Chemiker

Supporting Information

Global Aromaticity and Antiaromaticity in Porphyrin Nanoring Anions**

*Martin D. Peeks, Michael Jirasek, Timothy D. W. Claridge, and Harry L. Anderson**

anie_201909032_sm_miscellaneous_information.pdf

Contents

S1 Computational Chemistry S3
S1.1 Summary of NICS values S3
S2 Synthesis and sample preparation S4
S2.1 Quantification of $CoCp^*_2$ concentration S7
S3 NMR assignments S8
S3.1 Summary of assignments S8
S3.2 Full-scale ^{1}H spectra S9
S3.3 Proof of reversibility S10
S3.4 Additional spectra for hexa-anions S10
S3.5 Additional spectra for tetra-anions S18
S3.6 Dynamic NMR analyses S22
S3.6.1 Neutral ***c*-P6** S23
S3.6.2 ***c*-P6^{6-}** S25
S3.6.3 Summary S26
S4 Cartesian coordinates S27
S4.1 ***c*-P6** (B3LYP/6-31+G*) S27
S4.2 ***c*-P6^{4-}** (B3LYP/6-31+G*) S28
S4.3 ***c*-P6^{6-}** (B3LYP/6-31+G*) S29

List of Figures

S1 NICS(0)$_{zz}$ for ***c*-P6** in various oxidation states . . . S3
S2 NICS(0)$_{iso}$ for ***c*-P6** in various oxidation states, calculated perpendicular to the nanoring plane . . . S4
S3 Anisotropy of the induced current density and induced current vectors . . . S4
S4 Photographs of the experimental procedure for preparation of porphyrin nanoring anions . . . S6
S5 ^{1}H NMR spectrum for quantification of $CoCp^*_2$ using $FcPF_6$ . . . S7
S6 Full-scale versions of spectra shown in main text Figure 3 . . . S9
S7 Proof of reversibility of reduction of ***c*-P6·T6** . . . S10
S8 NMR spectra comparing ***c*-P6**$^{6-}$ and ***c*-P6·T6**$^{6-}$ . . . S10
S9 ^{1}H TOCSY of ***c*-P6·T6**$^{6-}$ showing o_i, o_o, and *p* resonances . . . S11
S10 ^{1}H TOCSY of ***c*-P6·T6**$^{6-}$ showing THS_i and THS_o multiplets . . . S12
S11 ^{1}H NOESY of ***c*-P6·T6**$^{6-}$ . . . S13
S12 ^{1}H–^{13}C HSQC of ***c*-P6·T6**$^{6-}$ . . . S14
S13 Diffusion-edited ^{1}H spectra for ***c*-P6·T6**$^{6-}$ . . . S15
S14 Diffusion-edited ^{1}H spectra for ***c*-P6**$^{6-}$ . . . S16
S15 NMR spectra comparing ***c*-P6·T6**$^{6+}$ and ***c*-P6·T6**$^{6-}$ . . . S17
S16 NMR spectra comparing ***c*-P6·T6**$^{4-}$ and ***c*-P6**$^{4-}$ . . . S18
S17 ^{1}H NOESY of ***c*-P6·T6**$^{4-}$ . . . S19
S18 ^{1}H NOESY of ***c*-P6**$^{4-}$ . . . S20
S19 Diffusion-edited ^{1}H spectra for ***c*-P6·T6**$^{4-}$ . . . S21
S20 NMR spectra comparing ***c*-P6·T6**$^{4+}$ and ***c*-P6·T6**$^{4-}$ . . . S22
S21 Schematic for dynamic NMR processes in ***c*-P6** . . . S22
S22 VT-NMR of ***c*-P6** (low temperature) . . . S23
S23 VT-NMR of ***c*-P6·T6** (high-temperature) . . . S24
S24 EXSY results for exchange of o_o and o_i in ***c*-P6·T6** . . . S25
S25 EXSY results for exchange of o_o and o_i in ***c*-P6**$^{6-}$ . . . S26

List of Tables

S1 Summary of NICS data . . . S3
S2 Summary of ellipticity of ***c*-P6** as a function of oxidation state and functional . . . S3
S3 Summary of ^{1}H NMR chemical shifts . . . S8
S4 Summary of dynamic NMR data . . . S26

S1 Computational Chemistry

DFT geometry optimizations were performed using the B3LYP, ωB97X-D, and M06-2X functionals and the 6-31+G* all-electron basis set,[S1–S8] as implemented in Gaussian16/A.03.[S9] The *meso*-aryl groups on the nanorings were truncated to –H. NICS surfaces were calculated with a 20 Å × 20 Å grid of ghost atoms with 1 Å spacing.

S1.1 Summary of NICS values

Table S1: NICS(0)$_{iso}$ and NICS(0)$_{zz}$ (all units ppm) at the centers of ***c*-P6** as a function of oxidation state and DFT functional. The basis set was 6-31+G* in all cases.

	Neutral		4−		6−	
	NICS(0)$_{iso}$	NICS(0)$_{zz}$	NICS(0)$_{iso}$	NICS(0)$_{zz}$	NICS(0)$_{iso}$	NICS(0)$_{zz}$
B3LYP	−1.51	1.00	88.69	267.85	−12.95	−39.53
ωB97X//B3LYP	−1.82	0.58	6.98	23.84	−15.10	−43.78
M06-2X//B3LYP	−1.81	0.64	11.46	37.07	−14.72	−43.69
ωB97X//ωB97X-D	−1.85	0.57	−0.65	1.20	−14.89	−43.20
M06-2X//M06-2X	−1.88	0.58	4.48	16.24	−14.34	−42.73

Table S2: Ellipticity of ***c*-P6** as a function of oxidation state as calculated by different functionals. The basis set was 6-31+G* in all cases. Ellipticity is calculated as the ratio of the lengths of the semi-minor and semi-major axes.

	Neutral	4−	6−
B3LYP	1.000	1.098	1.000
ωB97X-D	1.000	1.318	1.000
M06-2X	1.000	1.185	1.000

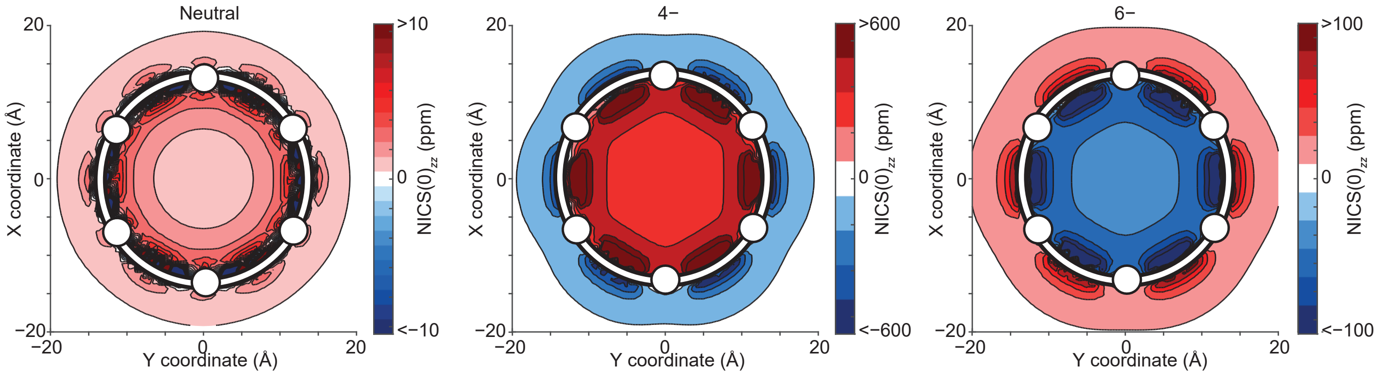

Figure S1: NICS(0)$_{zz}$ for ***c*-P6**, ***c*-P6^{4-}**, and ***c*-P6^{6-}**. Level of theory: B3LYP/6-31+G*. White circles indicate the location of porphyrin subunits; the white ring indicates the approximate circumference of the porphyrin nanoring.

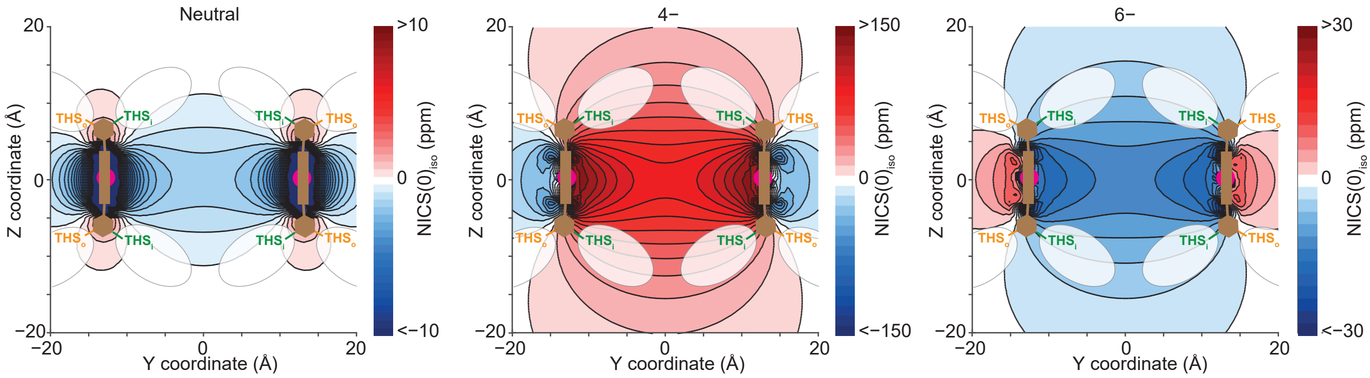

Figure S2: NICS(0)$_{iso}$ for (left–right) ***c*-P6**, ***c*-P6^{4-}**, and ***c*-P6^{6-}**, calculated in the *yz* plane (i.e. perpendicular to the nanoring plane). The approximate locations of the porphyrin subunits and their sidechains are shown. The white ovals denote the approximate region occupied by the solubilizing trihexylsilyl groups. In the 4− and 6− oxidation states, THS$_o$ is located in a region of no magnetic (de)shielding. Level of theory: B3LYP/6-31+G*.

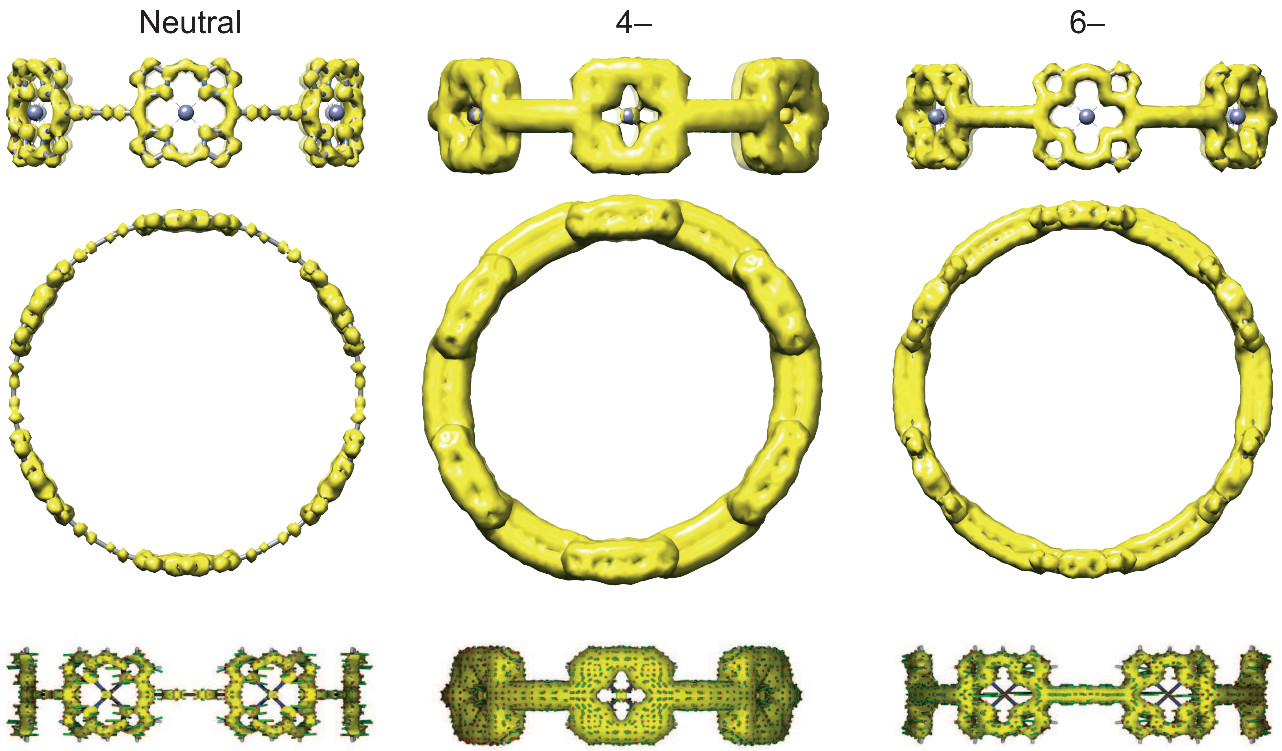

Figure S3: Top, middle: Anisotropy of the induced current density (AICD) for ***c*-P6**, ***c*-P6^{4-}**, and ***c*-P6^{6-}**; bottom: AICD and induced current vectors, for an applied magnetic field threading the ring from bottom-to-top. Level of theory: B3LYP/6-31+G*.

S2 Synthesis and sample preparation

***c*-P6** and ***c*-P6·T6** were prepared as described previously.[S10] The porphyrin subunits bore bulky trihexylsilyl (THS) solubilizing groups on their *meso*-aryl groups, to inhibit aggregation.

D_8-THF was purchased from Fluorochem (Eurisotop product) and was purified and dried by passage over freshly activated basic alumina, then degassed using the freeze-pump-thaw method, and finally stored over 4 Å molecular sieves. D_5-Pyridine ampoules were purchased from Fluorochem (Eurisotop product) and used without further purification, except freeze-pump-thaw degassing. D_6-Acetone was purchased from Sigma-Aldrich and used without further purification. $CoCp^*_2$ was purchased from Sigma-Aldrich and was purified by sublimation (200-250 °C under dynamic vacuum (*ca.* 2×10^{-1} mbar)), using a specialist piece of glassware (Figure S4). $CoCp^*_2$ was handled exclusively under argon.

Samples of ***c*-P6•T6** and ***c*-P6** were weighed out and transferred to J. Young tap Schlenk ampoules. The tube atmosphere was evacuated ($< 2 \times 10^{-1}$ mbar) and refilled with argon three times, and the nanoring samples were then dissolved in d_8-THF (+5% d_5-pyridine) to a concentration of, typically, 2.5 mg/mL and degassed by the freeze-pump-thaw method. Spectra could also be measured in neat d_8-THF. The effect of adding 5% d_5-pyridine was to make the spectra sharper, presumably owing to the increased polarity of the solvent mixture. This increase in solvent polarity may result in sharper peaks by causing changes in the dynamics of electron exchange with the free oxidizing agent. Alternatively, the sharper spectra could be a consequence of better solvation of the ionic species, leading to weaker ion pairing. A disposable Injekt syringe was used to transfer nanoring stock solution (500 µL) to a clean, dry, air-free J. Young NMR tube, situated in an NMR tube filler capped with a rubber Subaseal (Figure S4f).† The freshly-sublimated $CoCp_2^*$ was dissolved in d_8-THF and its concentration was determined as described below. For a single-shot reduction, an aliquot of $CoCp_2^*$ was added to the nanoring solution in the NMR tube, at −78 °C (dry ice/acetone bath), using a Hamilton or SGE Trajan syringe. We used long needles (>23 cm, e.g. ga26s/230mm/pst3 from Hamilton) to enable liquid transfer without tilting glassware. In general we have found that even 'non-coring' microsyringe needles have a tendency to introduce fragments of rubber from subaseals to solutions. We found it preferable to use a disposable 20G (or wider) needle to penetrate the septa, and then pass the microsyringe needle through the bore of this needle. This approach also avoided the inherent difficulties in penetrating a subaseal with a fine microsyringe needle. After addition of reducing agent, the J. Young NMR tube was capped under a strong flow of argon and its contents were freeze-pump-thaw degassed. **CAUTION:** argon condenses at liquid nitrogen temperatures. It is important to ensure that no condensed argon is present before an NMR tube is sealed and warmed to room temperature. This hazard can be avoided by using dry nitrogen instead of argon.

We also occasionally conducted reductions by addition of reducing agent to a frozen solution of nanoring in the NMR tube, then replacing the J. Young cap and applying vacuum to evaporate any condensed argon, and then continuing with the freeze-pump-thaw process. We emphasize, again, the inherent risks of using argon with liquid nitrogen.

All solvents and reagents were handled using standard Schlenk line techniques. Subaseals were used on all vessels wherever possible in order to avoid ingress of oxygen and water. A glove-box was not required for this work.

NMR spectra were recorded on a Bruker AVII 500 MHz, a Bruker AVIII 700 MHz, and a Bruker AVIII 950 MHz. Assignments were made with the help of TOCSY and NOESY analyses, and with diffusion-edited ^{1}H spectra. It was not possible to confidently assign any resonance to proton "a" of the porphyrins in the reduced nanorings. The broad resonances at −1.8 ppm in ***c*-P6**$^{6-}$ and ***c*-P6•T6**$^{6-}$ were attributable to decomposition: they grew in intensity over time and upon repeated addition of reducing agent, with consequent introduction of trace oxygen.

The spectrum of the 4− oxidation state of ***c*-P6•T6** is extremely sensitive to the amount of reducing agent added: when a slight excess or deficit of reducing agent is introduced (e.g., we estimate, 3.8 eq or 4.2 eq), broader THS resonances are observed and the "inner" THS resonances are more strongly deshielded and reduced in intensity. The spectra of both the 6− and 4− oxidation states are very sensitive to temperature: the sharpest ^{1}H spectrum of ***c*-P6•T6**$^{6-}$ is observed around 273 K, whereas the sharpest spectrum of ***c*-P6•T6**$^{4-}$ is observed around 288 K. Below these temperatures, there are signs of aggregation; above, spectra are broadened, presumably due to electron exchange.

†The NMR tube filler is a cylindrical piece of glass (i.d. approx 22 mm) equipped with a side arm tap and tapered glass tube (for attachment to the Schlenk line), and featuring at the upper part of the cylinder a female B24/29 joint (for insertion of a subaseal) and at the lower part of the cylinder a 5 mm thermometer adapter with a compression O-ring. Typically, NMR tubes can be evacuated to at least 5×10^{-2} mbar with this setup; small leaks in the O-ring part can be avoided by the application of a thin film of vacuum grease, or PTFE tape.

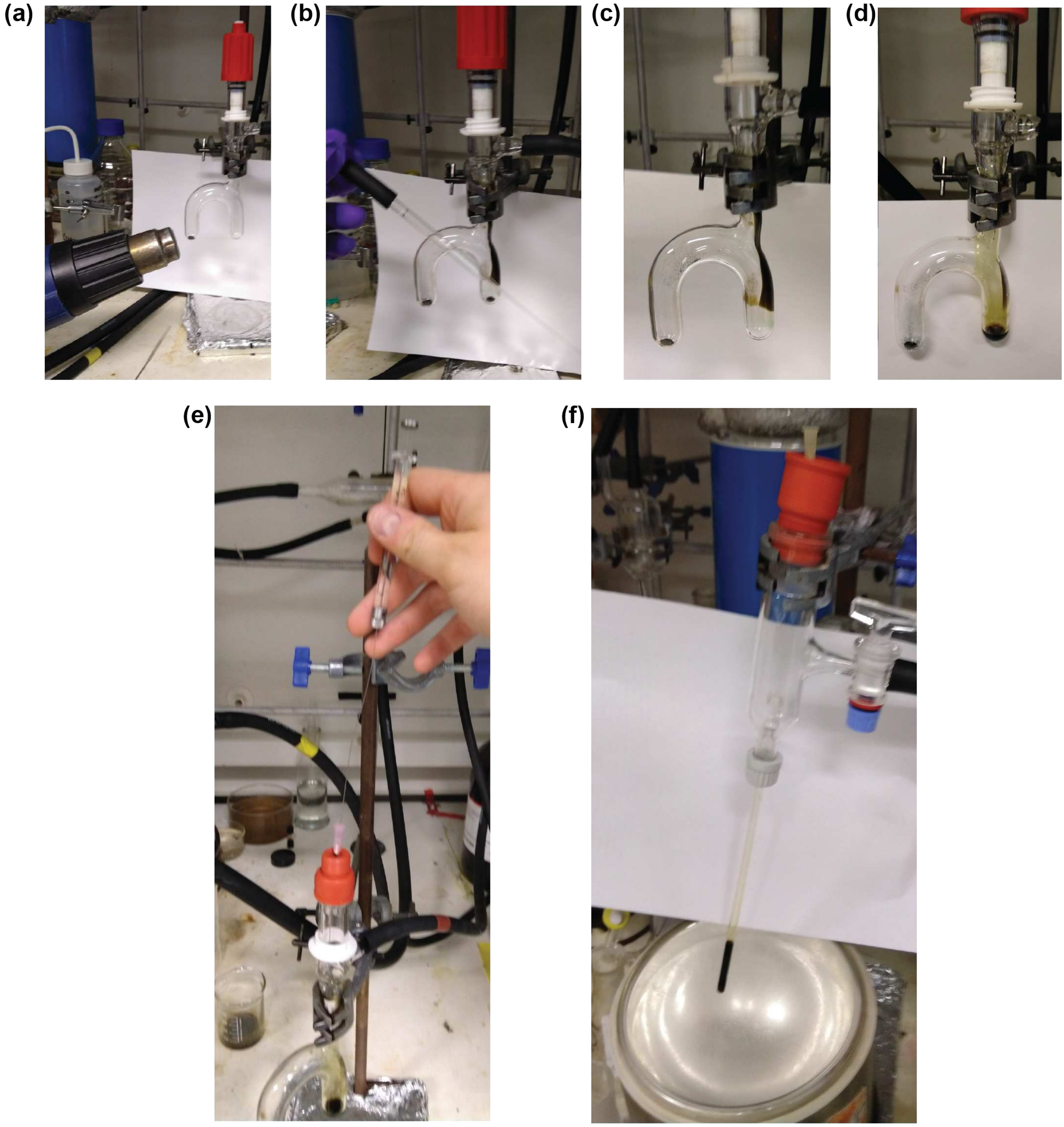

Figure S4: (a) Solid $CoCp^*_2$ is loaded into the left arm of the sublimation apparatus and is sublimated under vacuum using a heat gun; (b) after sublimation, pure $CoCp^*_2$ is deposited on the wall of the right arm of the apparatus, but some powder has also transferred to the right arm without sublimation. A Pasteur pipette is used to deliver a stream of dry nitrogen to remove the loose powder; (c) the right arm now only contains sublimated material; (d) addition of d_8-THF affords the stock solution of reducing agent; (e) a Hamilton syringe with a long needle is used to extract an aliquot of reducing agent. Note that the Hamilton needle is threaded through a pink (18G) needle; (f) the reducing agent is then added to the NMR tube in the NMR tube filler, with cooling. Note the presence of a cream (19G) needle in the subaseal, through which the Hamilton needle will pass.

For the ring without template, the sharpest spectra are observed at much lower temperatures: 223 K for 6− and 213 K for 4−.

The 6− oxidation state is stable in solution for 2 to 3 days at moderately reduced temperature (~273 K), or up to 1 day at room temperature. The 4− oxidation state is noticeably less stable: the sample corresponding to the NMR spectrum shown in main text Figure 3 exhibited signs of decomposition over the course of hours at 288 K (the temperature of NMR analysis, at which the sharpest spectra are observed). Samples of the reduced rings without templates are much more stable during NMR analyses, probably owing to the lower temperature used.

S2.1 Quantification of $CoCp^*_2$ concentration

The concentration of $CoCp^*_2$ solutions was estimated by quenching an aliquot (typically 20 µL) of $CoCp^*_2$ stock solution (in d_8-THF) by addition to an excess of solid ferrocenium hexafluorophosphate in a dry, air-free, NMR tube. The addition was conducted under an atmosphere of argon, using the NMR tube filler. The d_8-THF was removed by application of dynamic vacuum and the residue was redissolved in d_6-acetone (500 µL), to ensure complete dissolution of the mixture. An aliquot of tert-butyl benzene (2.5 µL, Alfa Aesar) was added as an internal reference. The ^{1}H NMR spectrum was recorded with sufficiently long delay time for quantitation ($d_1 = 5$ sec) and the relative integrals of the internal reference and the CH_3 peak of decamethylcobaltocenium were determined by deconvolution of the relevant resonances.

The typical concentration of $CoCp^*_2$ was 1 mM when used for stoichiometric reduction (*i.e.* to reach the 4− oxidation state). Higher concentrations (*ca* 18 mM) were used when addition of excess reducing agent was desired (*i.e.* to reach the 6− oxidation state).

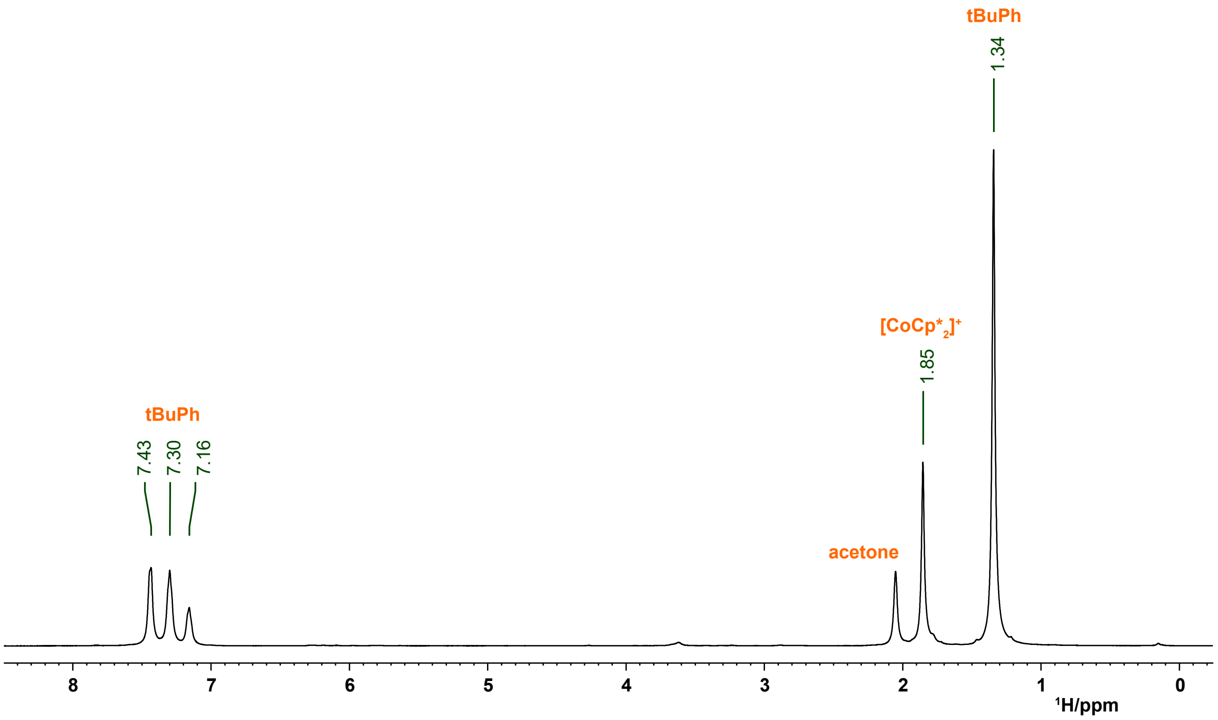

Figure S5: ^{1}H NMR (500 MHz, 298 K, d_6-acetone) spectrum of a mixture of $CoCp^*_2$, $FcPF_6$, and tBuPh.

S3 NMR assignments

S3.1 Summary of assignments

Table S3: Summary of ^{1}H NMR chemical shifts (ppm) for ***c*-P6·T6** in its neutral (500 MHz, 298 K, d_8-THF + 5% pyridine), 4− (500 MHz, 288 K, d_8-THF + 5% pyridine), and 6− (950 MHz, 273 K, d_8-THF + 5% pyridine) oxidation states. ^{13}C chemical shifts for the adjacent carbon are given in parentheses where available. The chemical shift difference between inner (δ_i) and outer (δ_o) protons is given by: $\Delta\delta = \delta_i - \delta_o$

Proton(s)	δ_H neutral	δ_H 4−	δ_H 6−
α	2.35	12.8–15.5	5.80
β	5.19	12.8–15.5	4.4–4.1
γ	5.57	12.8–15.5	4.4–4.1
δ	5.63	12.8–15.5	4.4–4.1
a	9.59	–	3.02?
b	8.78	17.64	3.75
o_o	8.16	6.77	7.79
o_i	8.36	12.18	4.44
p	8.09	9.17	6.73 (137.4)
THS_o CH_3	0.8–1.6	0.7–1.9	0.80 (14.2)
THS_i CH_3	0.8–1.6	2.5–3.9	−0.38 (13.1)
THS_o $SiCH_2$	0.8–1.6	0.7–1.9	0.72 (12.5)
THS_i $SiCH_2$	0.8–1.6	2.5–3.9	−0.87 (10.9)
$\Delta\delta_{THS\ CH_3}$	*ca.* 0	*ca.* 2	−1.18 (−1.1)
$\Delta\delta_{THS\ SiCH_2}$	*ca.* 0	*ca.* 2	−1.59 (−1.6)
$\Delta\delta_{ortho}$	0.20	5.41	−3.35

S3.2 Full-scale ^{1}H spectra

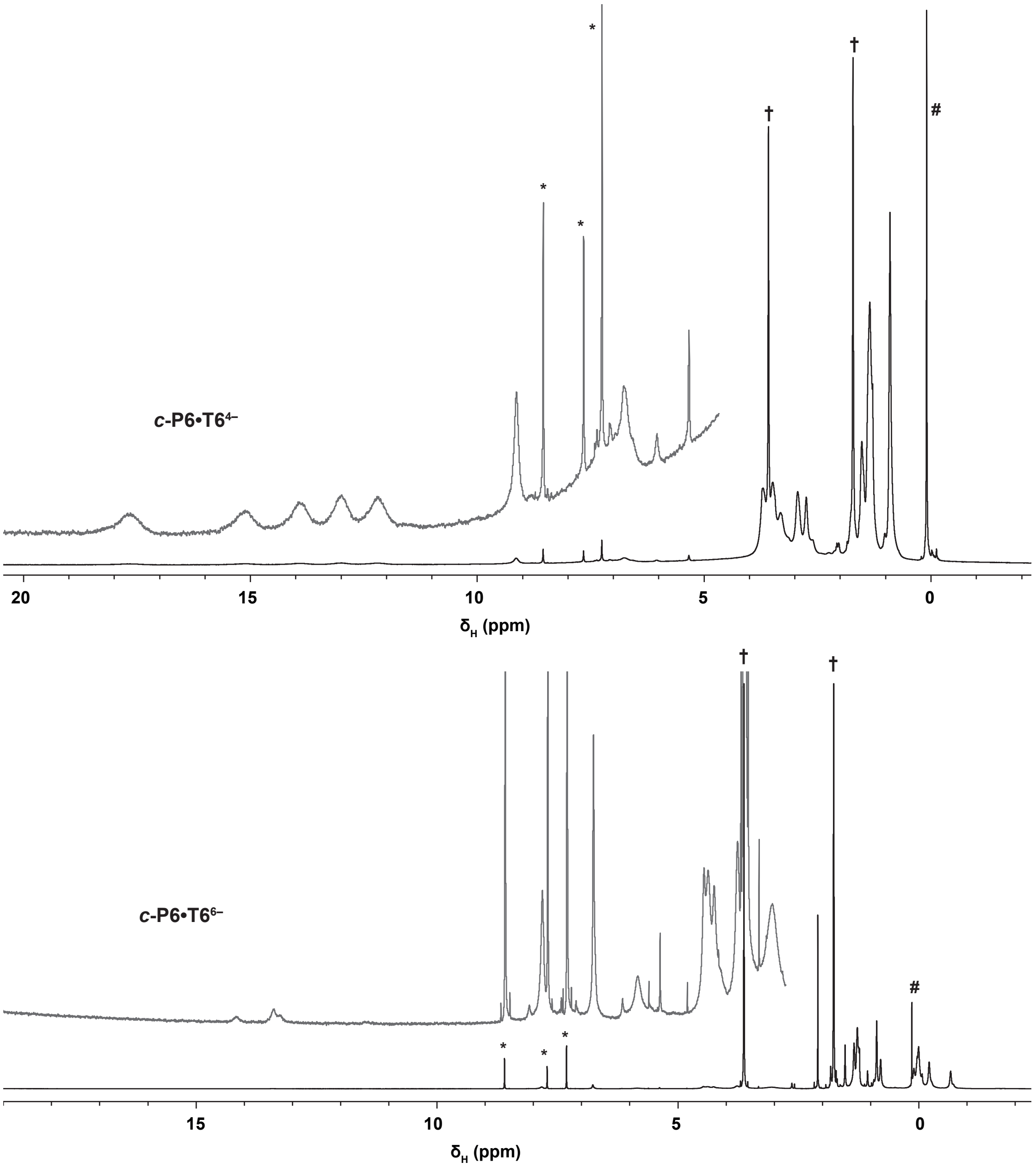

Figure S6: Full-scale versions of the spectra shown in main text Figure 3. ***c*-P6•T6**$^{4-}$ (500 MHz, 288 K, d_8-THF + 5% d_5-pyridine); ***c*-P6•T6**$^{6-}$ (950 MHz, 273 K, d_8-THF + 5% d_5-pyridine). In the expansions shown in grey, tall peaks are truncated. Impurities are denoted by symbols: * is pyridine; † is THF; # is grease.

S3.3 Proof of reversibility

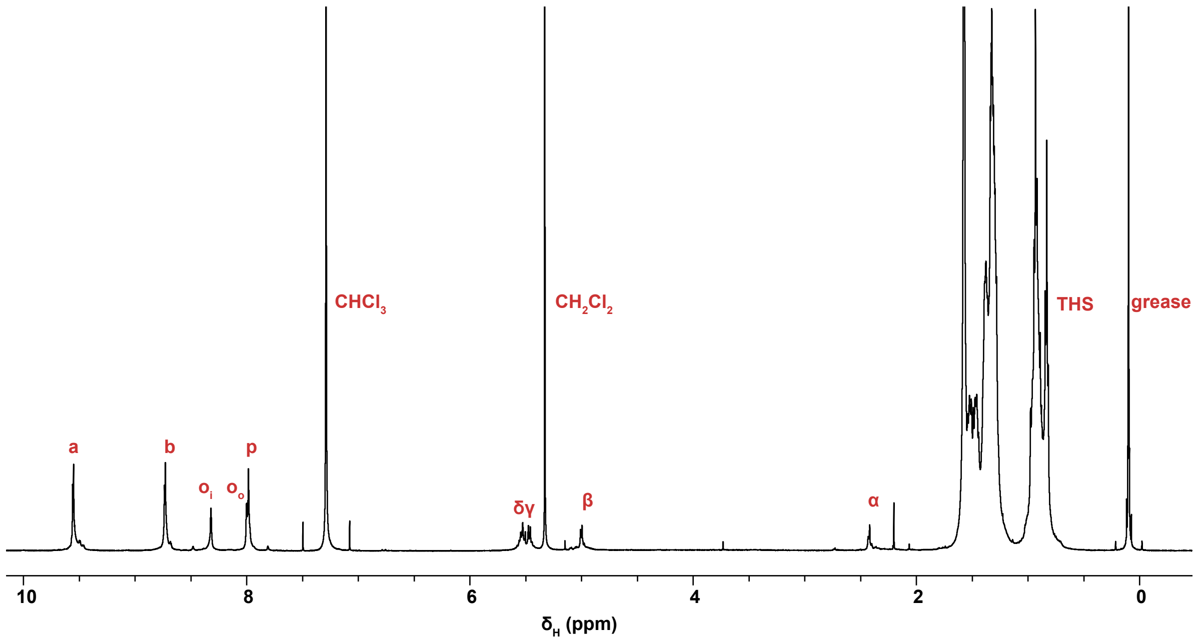

Figure S7: *c*-**P6·T6** (500 MHz, 298 K, $CDCl_3$) after reduction to the 6− oxidation state and subsequent re-oxidation using $FcPF_6$, and passage through an alumina plug ($CHCl_3$), and then through a size-exclusion column (Biobeads SX-1, toluene).

S3.4 Additional spectra for hexa-anions

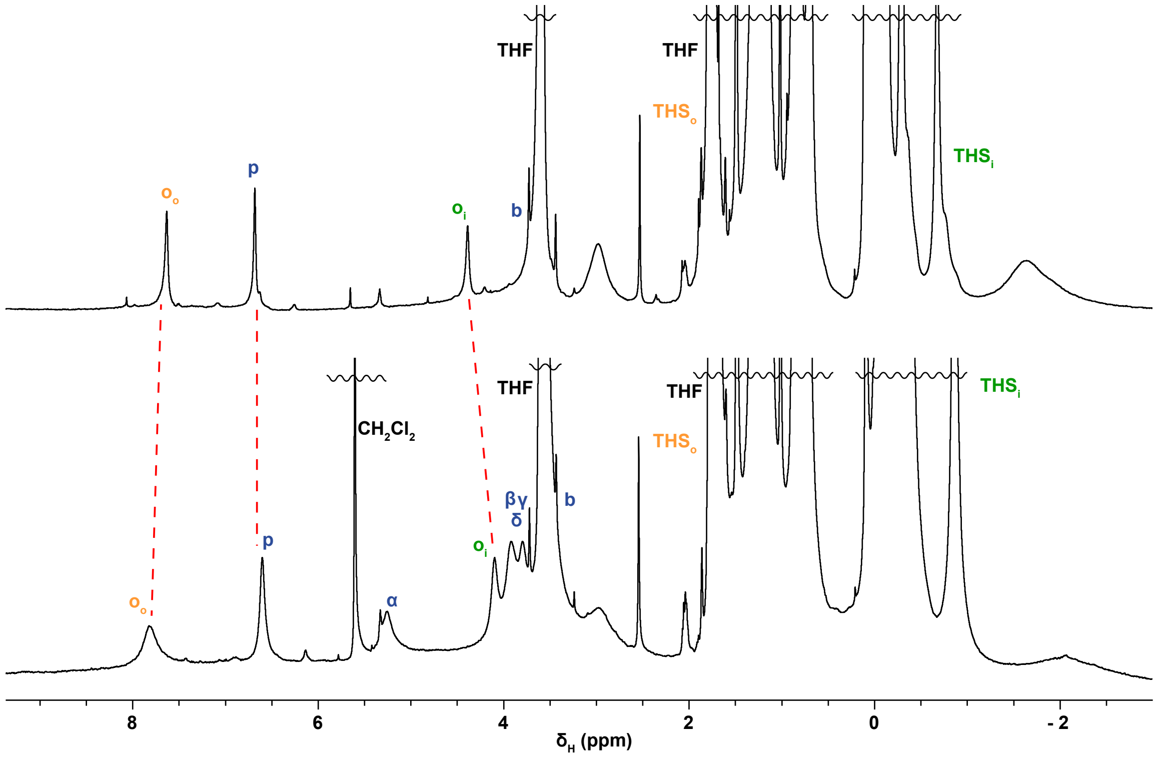

Figure S8: Comparison of ^{1}H NMR spectra of *c*-**P6**$^{6-}$ (top, 228 K) and *c*-**P6·T6**$^{6-}$ (bottom, 248 K) (both 500 MHz, d_8-THF). In these spectra, the resonance labelled **b** overlaps with the THF resonance at 3.58 ppm.

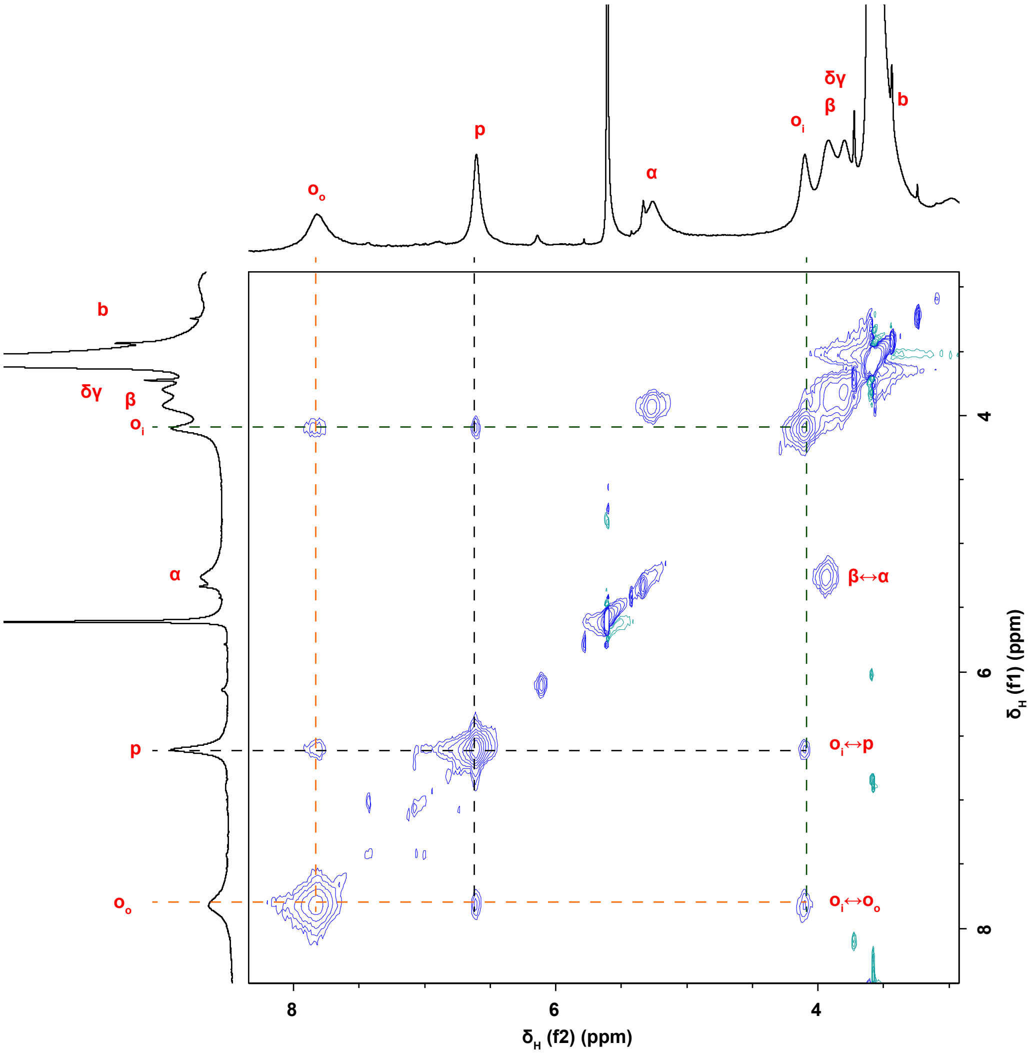

Figure S9: ^{1}H TOCSY spectrum of ***c*-P6•T6**$^{6-}$ (500 MHz, 248 K, d_8-THF). This region shows the correlations between the o_i, o_o, and p resonances, which confirms their assignment since there is no other AXY spin system in the molecule. $t_{\text{mix}} = 80$ ms.

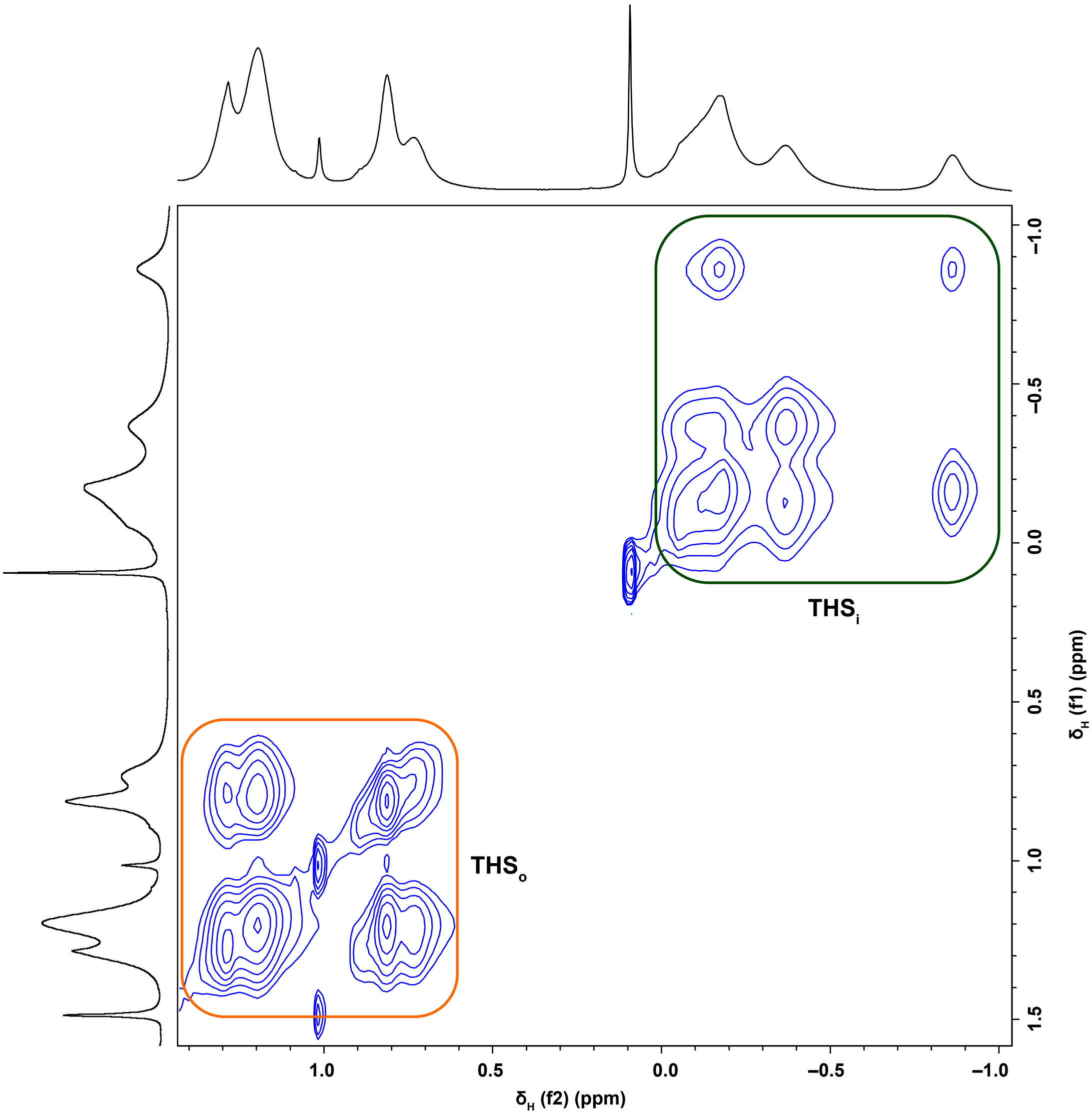

Figure S10: ^{1}H TOCSY spectrum of ***c*-P6·T6**$^{6-}$ (500 MHz, 248 K, d_8-THF). This region shows the correlations within the *THS$_i$* and *THS$_o$* multiplets, respectively. $t_{mix} = 80$ ms.

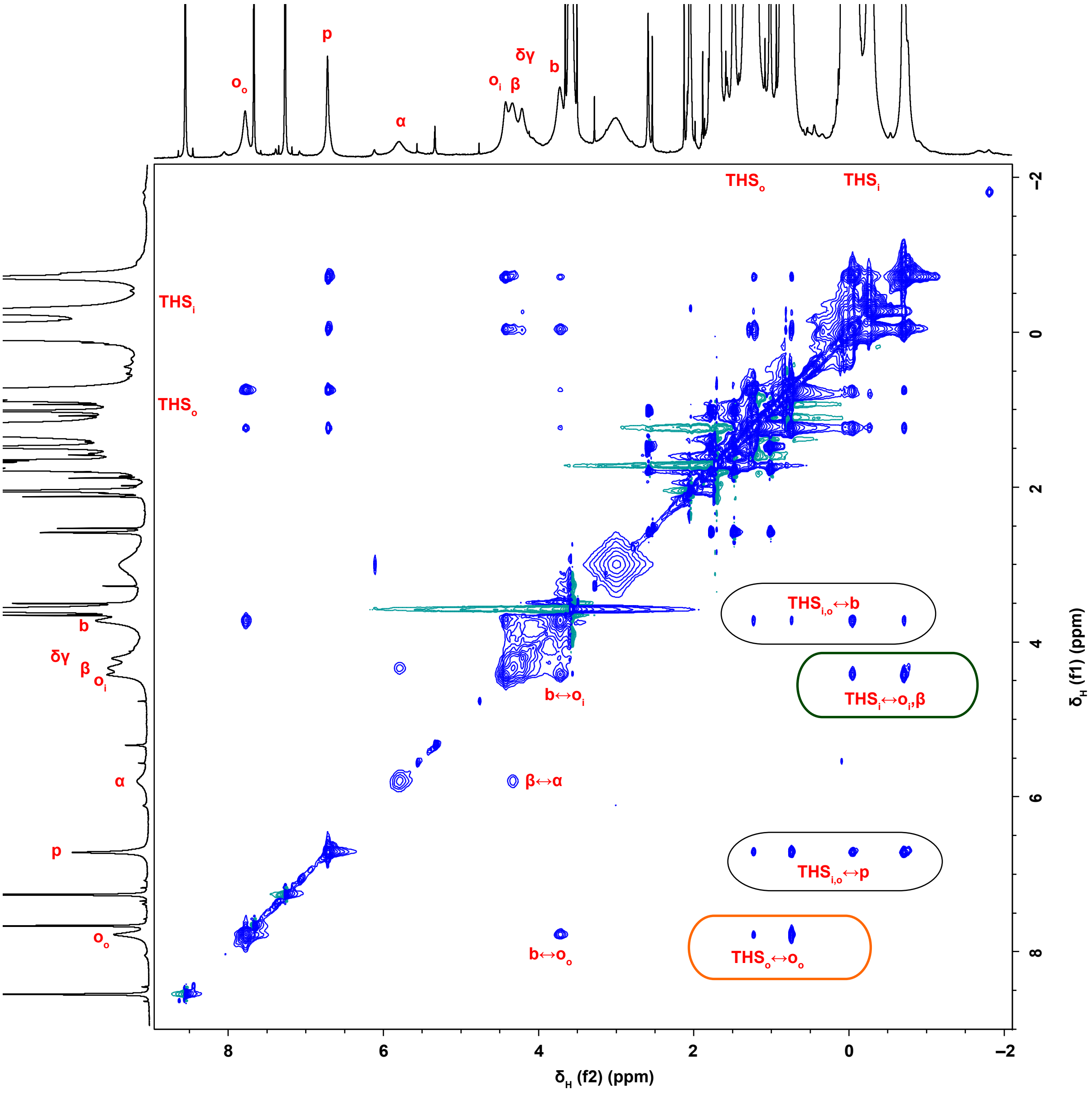

Figure S11: ^{1}H NOESY spectrum of ***c*-P6·T6**$^{6-}$ (950 MHz, 273 K, d_8-THF + 5% d_5-pyridine). There are no visible cross-peaks outside the region shown. t_{mix} = 300 ms.

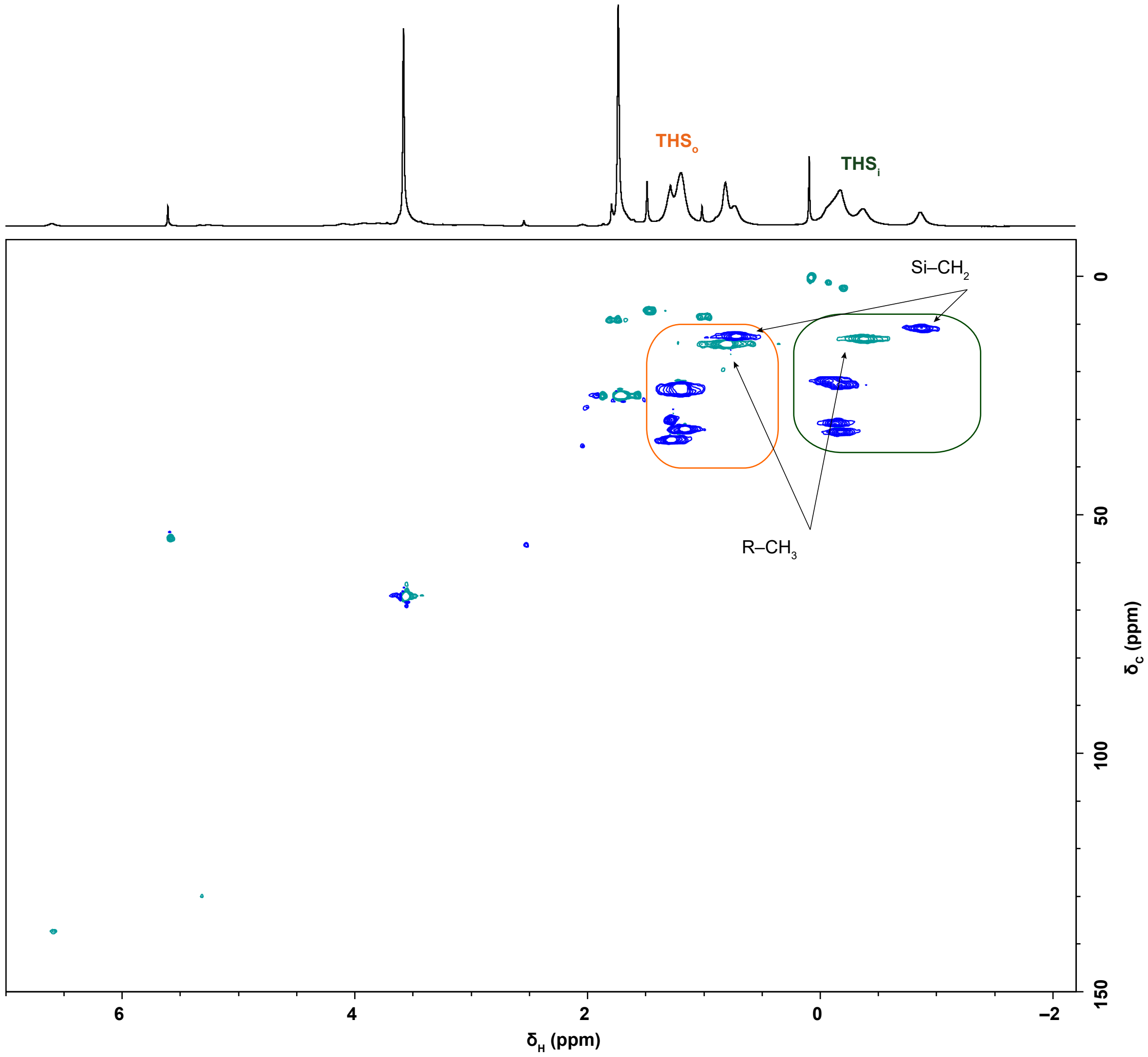

Figure S12: ^{1}H–^{13}C HSQC of ***c*-P6·T6**$^{6-}$ (500 MHz, 248 K, d_8-THF). There are no visible cross-peaks outside the region shown.

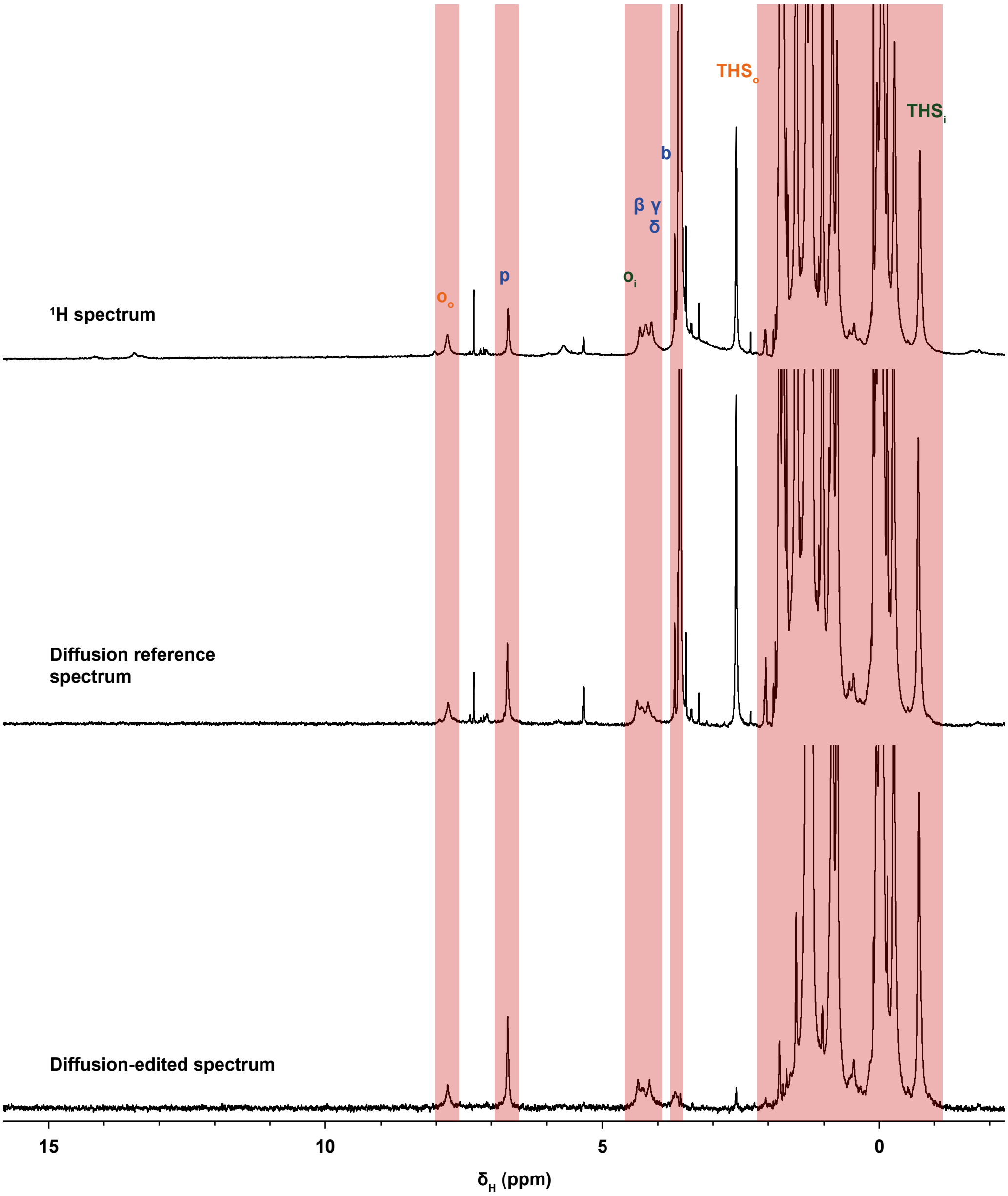

Figure S13: Diffusion edited spectrum of ***c*-P6•T6**$^{6-}$ (700 MHz, 273 K, d_8-THF). Top: normal ^{1}H spectrum; middle: spectrum measured using diffusion-editing pulse sequence (double stimulated echo (DSTE) with $\Delta = 75$ ms and $\delta = 2000\,\mu$s), with 2% gradient power; bottom: spectrum measured using diffusion-editing pulse sequence (as above) with 90% gradient power. Resonances corresponding to slowly-diffusing components, consistent with ***c*-P6•T6**, are highlighted in red. Very broad resonances are attenuated by the diffusion pulse sequence due to rapid transverse relaxation and are therefore absent from both the diffusion reference spectrum and the diffusion-edited spectrum.

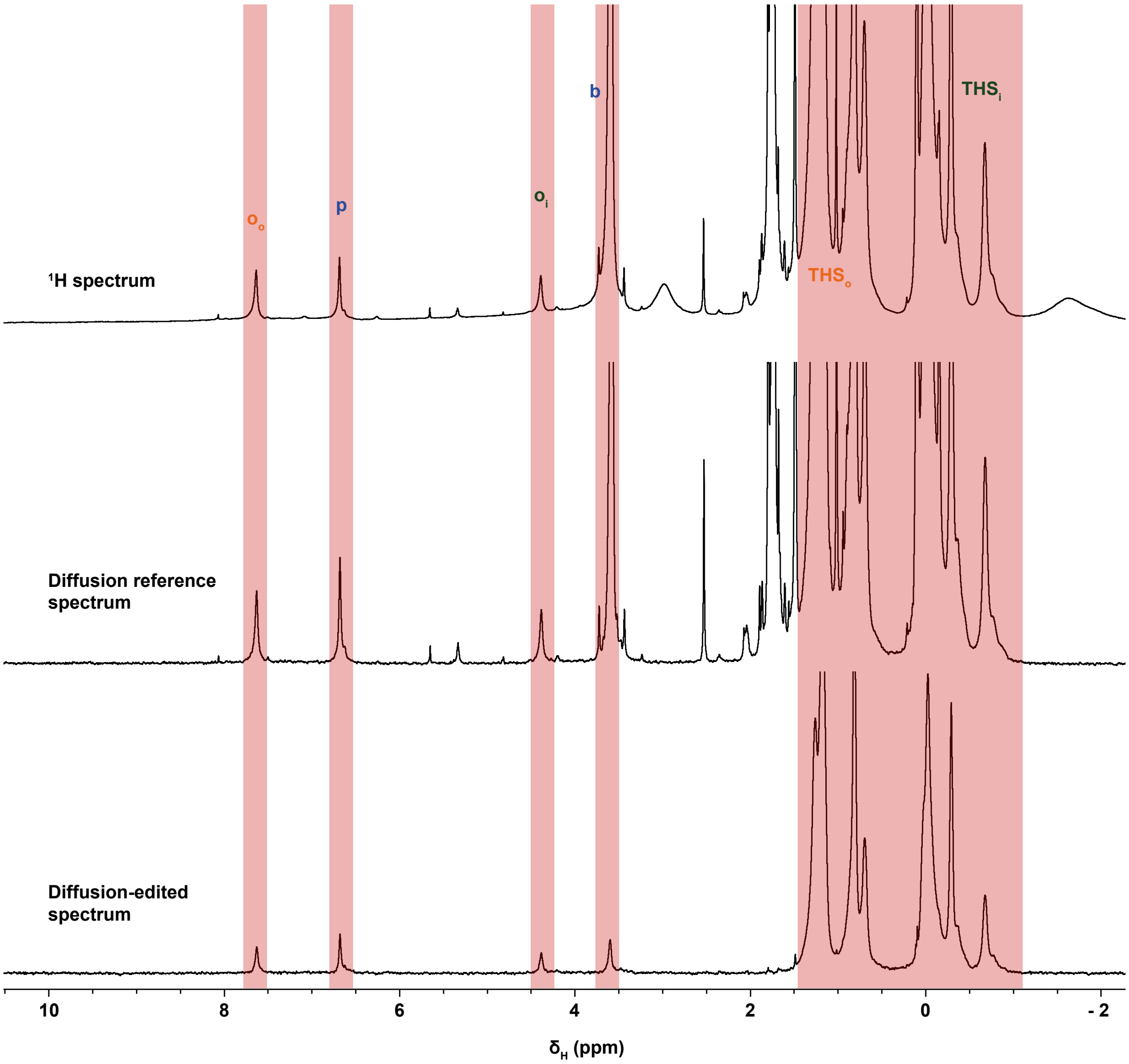

Figure S14: Diffusion edited spectrum of ***c*-P6**$^{6-}$ (500 MHz, 228 K, d_8-THF). Top: normal ^{1}H spectrum; middle: spectrum measured using diffusion-editing pulse sequence (double stimulated echo (DSTE) with $\Delta = 125$ ms and $\delta = 3200\,\mu$s), with 2% gradient power; bottom: spectrum measured using diffusion-editing pulse sequence (as above) with 90% gradient power. Resonances corresponding to slowly-diffusing components, consistent with ***c*-P6**, are highlighted in red. Very broad resonances are attenuated by the diffusion pulse sequence due to rapid transverse relaxation and are therefore absent from both the diffusion reference spectrum and the diffusion-edited spectrum.

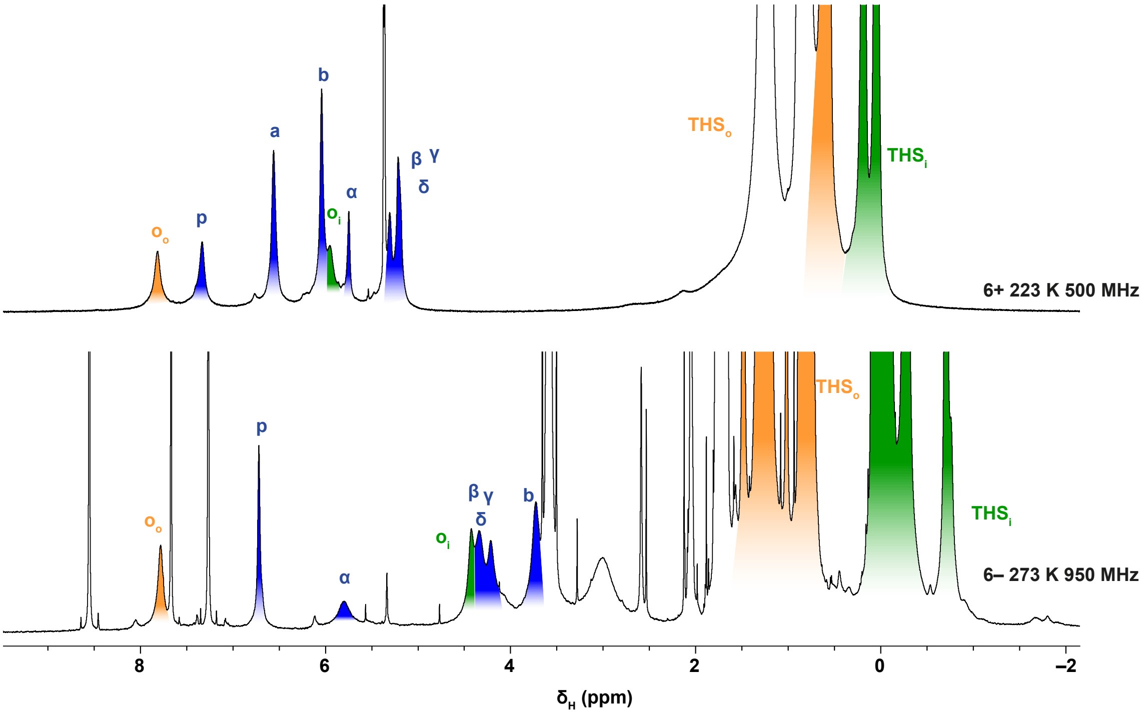

Figure S15: Comparison of the ^{1}H NMR spectra of ***c*-P6·T6**$^{6+}$ (CD_2Cl_2) and ***c*-P6·T6**$^{6-}$ (d_8-THF+ 5% pyridine). The spectrum for ***c*-P6·T6**$^{6+}$ has previously been reported.[S11]

S3.5 Additional spectra for tetra-anions

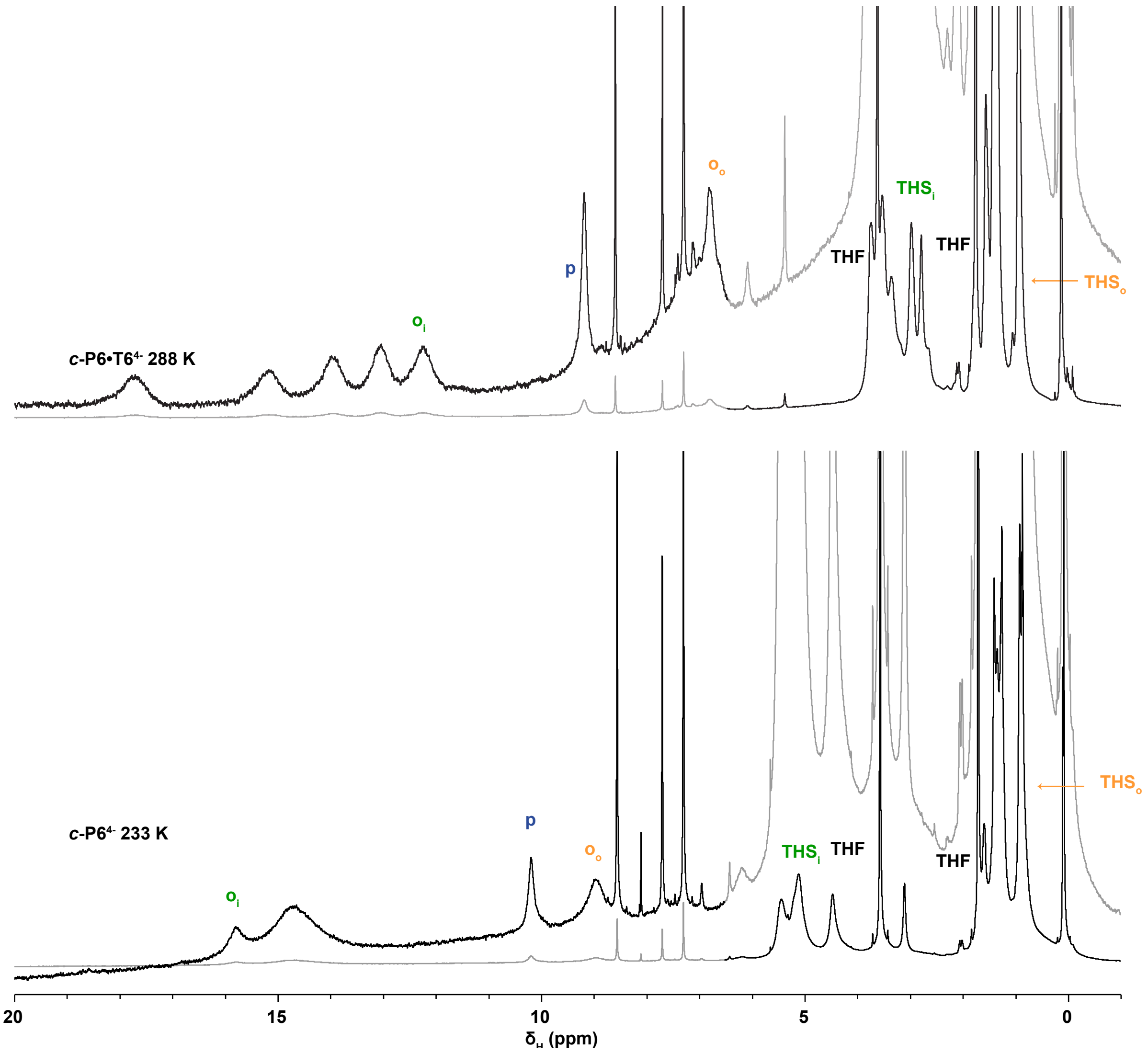

Figure S16: Comparison of ^{1}H NMR spectra of ***c*-P6·T6**$^{4-}$ (top, 288 K) and ***c*-P6**$^{4-}$ (bottom, 233 K) (both 500 MHz, d_8-THF + 5% d_5-pyridine). The large chemical shift differences for assigned resonances between the spectra with and without template are attributed to (a) temperature effects, since the spectra are measured at very different temperatures; (b) exchange with the 3− and 5− oxidation states, owing to slightly different extents of reduction in each sample.

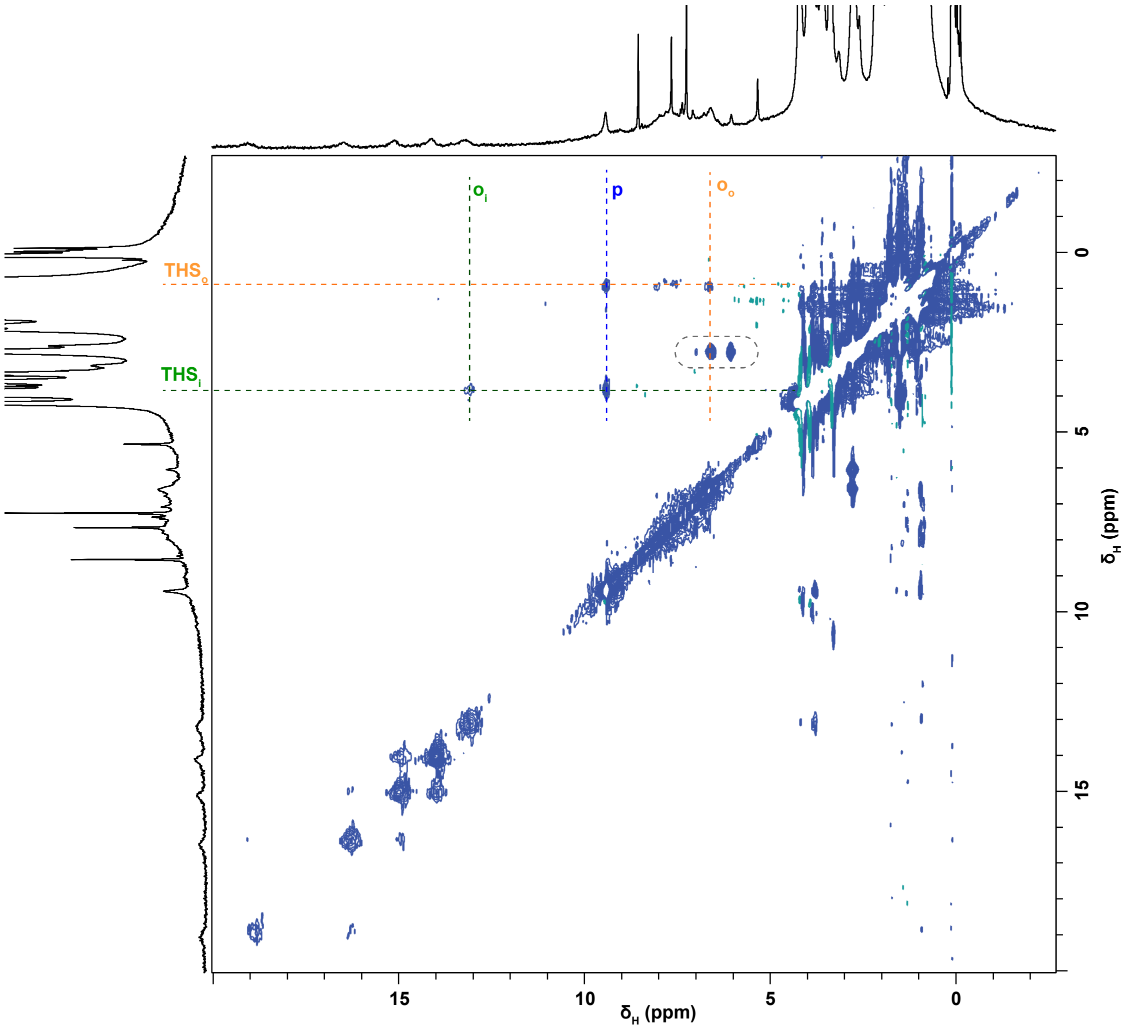

Figure S17: ^{1}H NOESY spectrum of ***c*-P6•T6^{4-}** (500 MHz, 288 K, d_8-THF + 5% d_5-pyridine). There are no visible cross-peaks outside the region shown. t_{mix} = 350 ms. Assignments are shown. The cross-peaks indicated by the grey oval correspond to impurities.

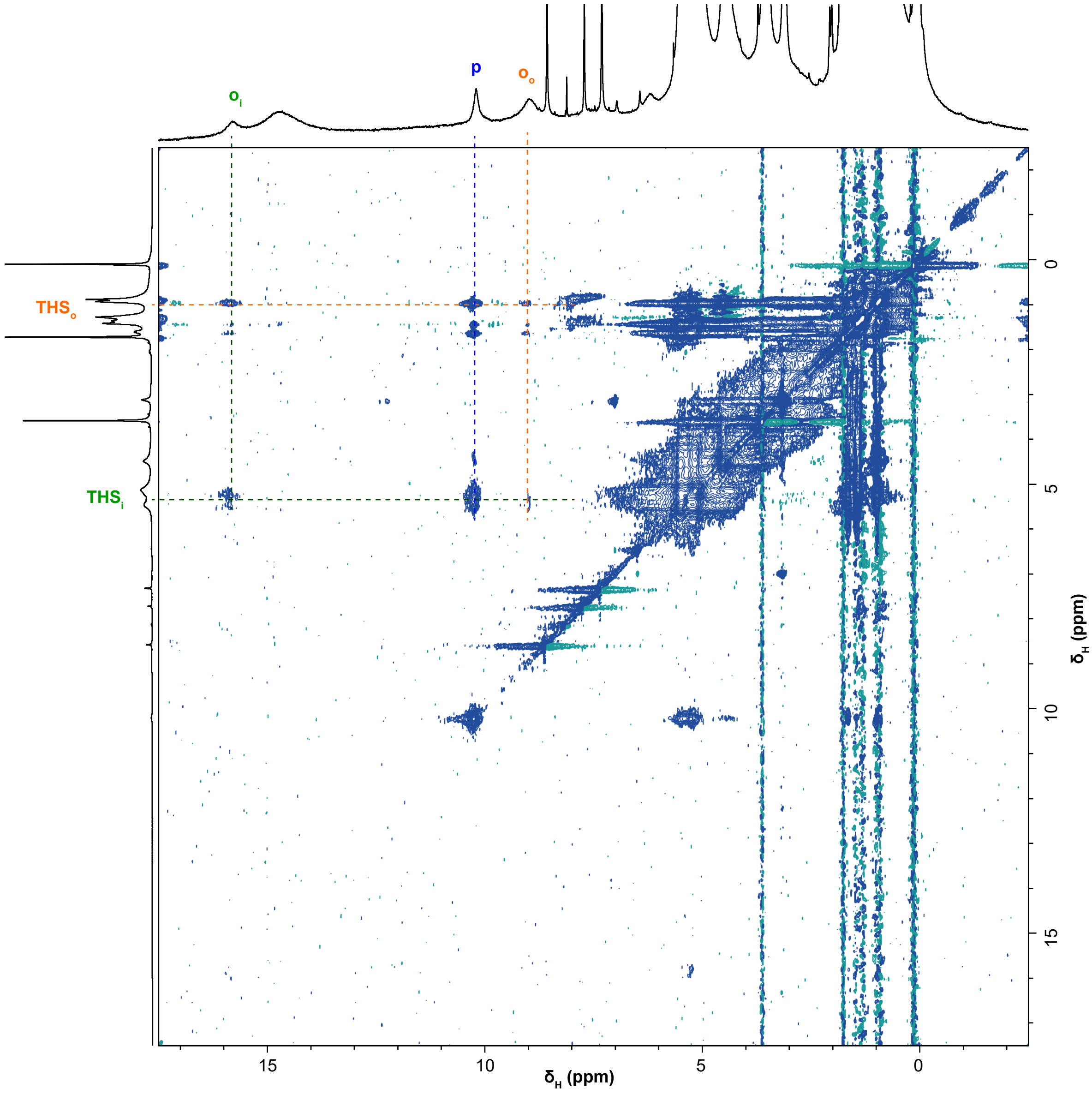

Figure S18: ^{1}H NOESY spectrum of ***c*-P6**$^{4-}$ (500 MHz, 233 K, d_8-THF + 5% d_5-pyridine). There are no visible cross-peaks outside the region shown. t_{mix} = 300 ms. Assignments are shown. Due to the absence of template, there is exchange between the inner and outer environments (i.e. $o_o \rightleftharpoons o_i$ and $THS_o \rightleftharpoons THS_i$), hence correlations appear between (for example) THS_i and o_o, and between THS_i and THS_o.

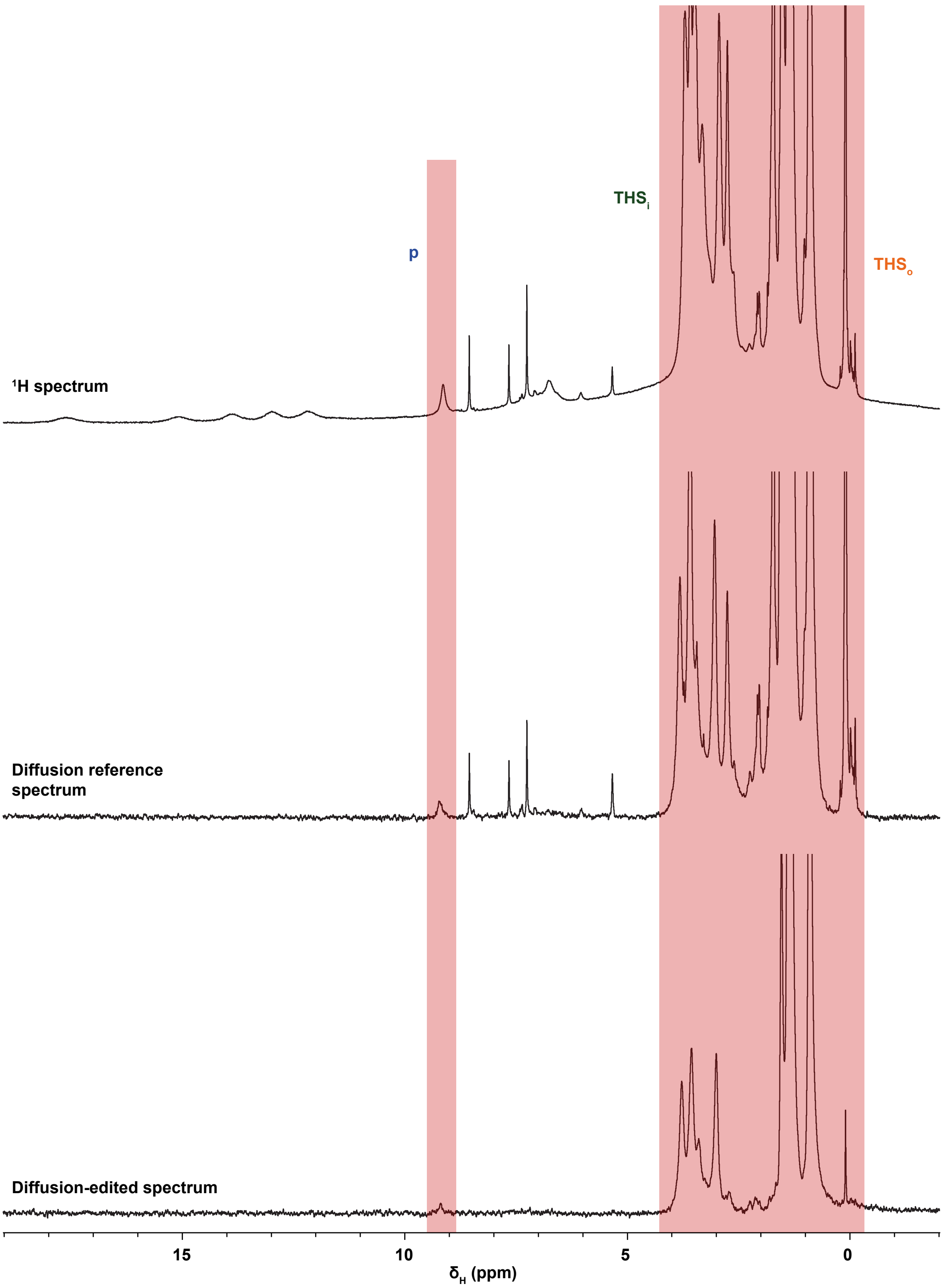

Figure S19: Diffusion edited spectrum of ***c*-P6•T6**$^{4-}$ (500 MHz, 288 K, d_8-THF + 5% d_5-pyridine). Top: normal ^{1}H spectrum; middle: spectrum measured using diffusion-editing pulse sequence (double stimulated echo (DSTE) with $\Delta = 75$ ms and $\delta = 2000\,\mu$s), with 5% gradient power; bottom: spectrum measured using diffusion-editing pulse sequence (as above) with 90% gradient power. Resonances corresponding to slowly-diffusing components, consistent with ***c*-P6•T6**, are highlighted in red. Very broad resonances are attenuated by the diffusion pulse sequence due to rapid transverse relaxation and are therefore absent from both the diffusion reference spectrum and the diffusion-edited spectrum.

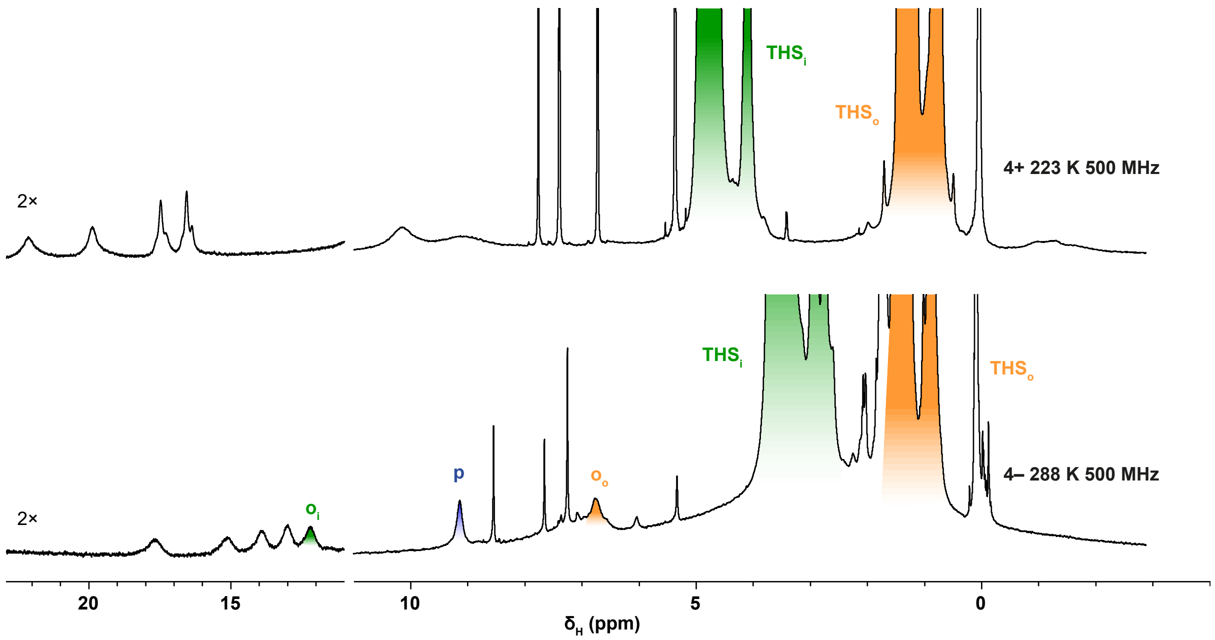

Figure S20: Comparison of the ^{1}H NMR spectra of ***c*-P6•T6**$^{4+}$ and ***c*-P6•T6**$^{4-}$. The spectrum for ***c*-P6•T6**$^{4+}$ has previously been reported.[S11]

S3.6 Dynamic NMR analyses

The **T6** template plays an important role in restricting inter-porphyrin rotation in ***c*-P6•T6**. In its absence, the neutral ring exhibits rapid inter-porphyrin rotation (φ_A in Figure S21), such that only one resonance for the o_o and o_i protons, in fast exchange, is observed in the ^{1}H NMR.

Evaluating the barrier to the φ_A exchange process permits its comparison between neutral and charged (aromatic) rings. An increase in torsion barrier could arise from increased aromatic stabilization energy.

In these analyses, it is important to separate the contribution of a second $o_o \rightleftharpoons o_i$ exchange process, which occurs even in the presence of **T6**. This exchange occurs as a result of the *meso*-aryl rotation, which is expected to be relatively hindered (φ_B in Figure S21). In the results below, the *meso*-aryl rotation φ_B is fully characterized by VT-NMR and EXSY for neutral ***c*-P6•T6**, explored by EXSY for ***c*-P6•T6**$^{6-}$, and the interporphyrin torsion φ_A is explored by VT-NMR for neutral ***c*-P6**, and by EXSY for ***c*-P6**$^{6-}$.

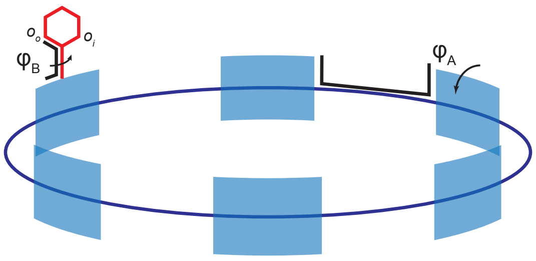

Figure S21: Schematic for the two mechanisms for exchange of o_o and o_i considered in this section. φ_A corresponds to rotation of the entire porphyrin subunit, thus exchanging o_o and o_i, whereas φ_B corresponds to rotation of the *meso*-aryl group, with the same result. The latter process occurs much more slowly.

S3.6.1 Neutral *c*-P6

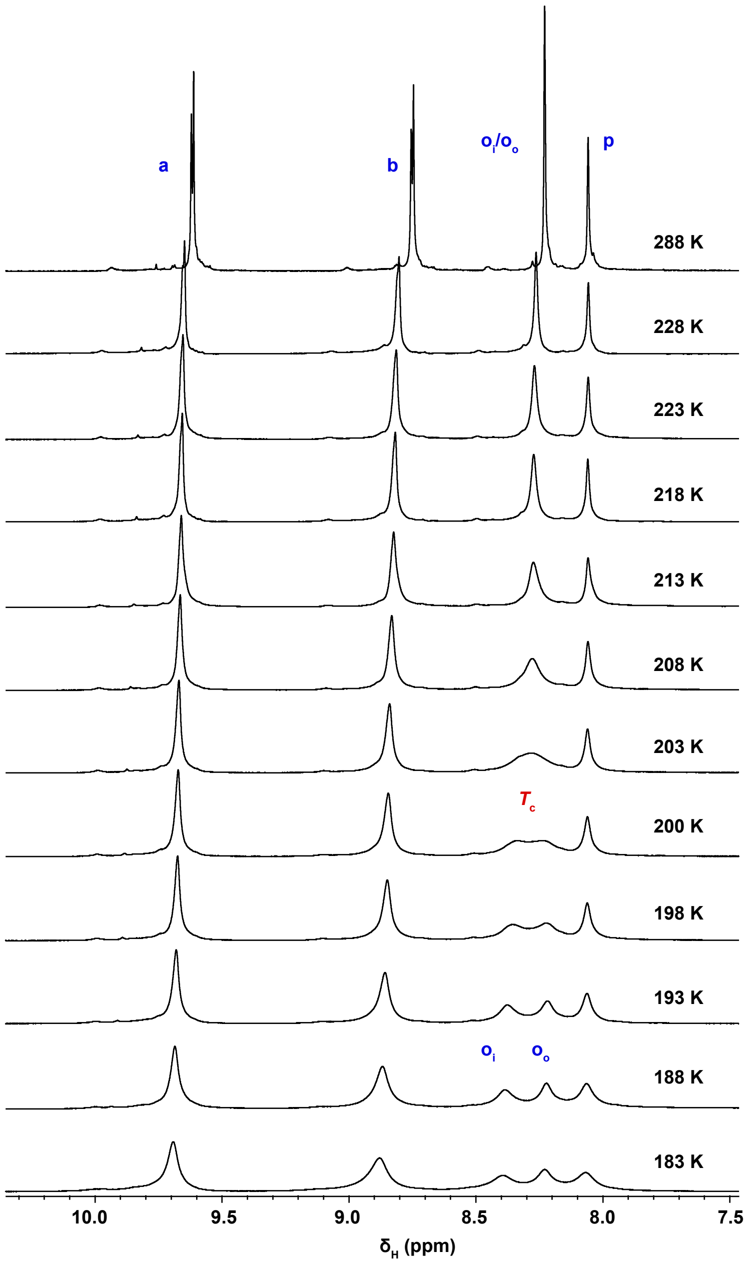

Figure S22: VT-NMR (500 MHz, d_8-THF) of ***c*-P6**. The coalescence point for the inner and outer *ortho* protons is indicated with T_c, 200-203 K.

At coalescence temperature $T_c = 200 - 203\,\text{K}$ (Figure S22), the chemical exchange rate constant for the exchange between o_o and o_i $k_{\text{chem}} = 577\,\text{s}^{-1}$, according to:[S12]

$$k_{\text{chem}} = 2 \cdot \frac{\pi \delta \nu}{\sqrt{2}} \tag{S1}$$

where $\delta\nu = 130\,\text{Hz}$, based on the chemical shift difference between o_o and o_i in ***c*-P6•T6** (0.26 ppm). Using the Eyring equation, $\Delta G^{\ddagger}_{200-203\,\text{K}} = (38.0 \pm 0.3)\,\text{kJ mol}^{-1}$.

The exchange of o_o and o_i arises through rotation of the entire porphyrin subunit, not through rotation of the *meso*-aryl group. We were able to confirm this by performing EXSY experiments on ***c*-P6•T6** at 298 K, which revealed negligible exchange between the o_o and o_i resonances. Upon heating a sample of ***c*-P6•T6** in d_8-toluene to 373 K, some line-broadening of the o_i and o_o resonances was observed but coalescence was not reached (Figure S23). EXSY

experiments at 353 K revealed $k_{chem} = 13\,s^{-1}$, corresponding to $\Delta G^{\ddagger}_{353\,K} = 79.4\,kJ\,mol^{-1}$ (Figure S24). Therefore we can safely conclude that the *meso*-aryl rotation does not affect the data shown in Figure S22.

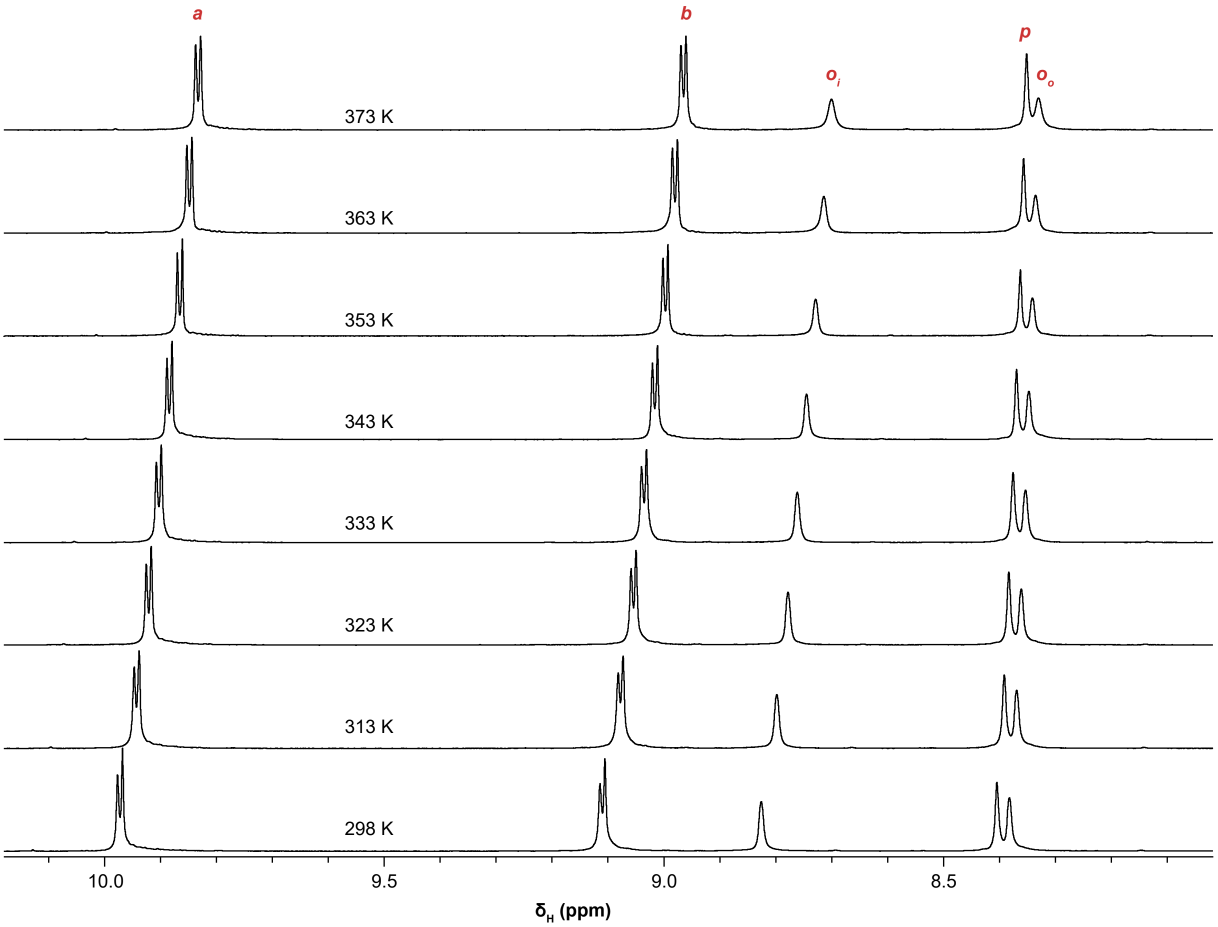

Figure S23: VT-NMR of *c*-**P6·T6** (500 MHz, d_8-toluene).

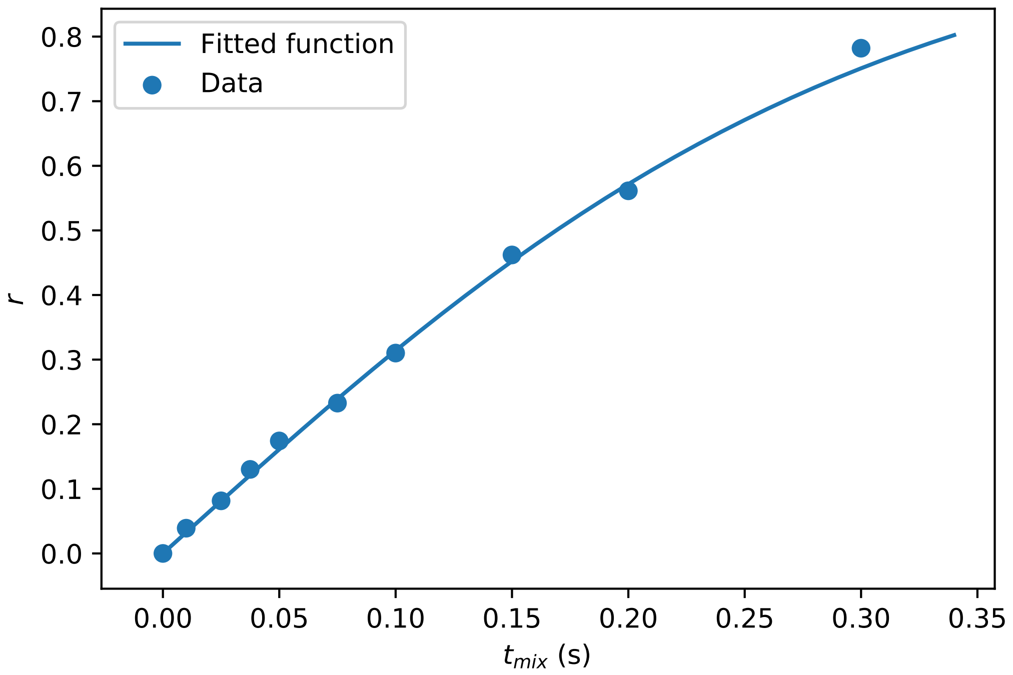

Figure S24: Experimental ratio of exchange intensities for the o_o and o_i resonances as a function of mixing time t_{mix} in neutral ***c*-P6•T6**. The o_i resonance was selectively excited. ***c*-P6•T6** (500 MHz, 353 K, d_8-toluene). The line represents the fit of Equation S5, with $k_{\mathrm{chem}} = 13\,\mathrm{s}^{-1}$
.

S3.6.2 *c*-P6^{6-}

The rate constant for exchange of the o_i and o_o protons in ***c*-P6^{6-}** was measured using a series of 1D-EXSY experiments with variable mixing times t_{mix} from 5 ms to 100 ms. Spectra were measured at 228 K on an Avance AVII 500 MHz spectrometer. The o_o resonance was selectively excited (7.7 ppm).

The system was modelled as a two-state exchanging system:

$$\mathrm{A} \underset{}{\overset{k_{\mathrm{chem}}}{\rightleftharpoons}} \mathrm{B}$$

with equal populations of A and B (i.e. outer and inner *ortho* protons, respectively). The intensities can be expressed as follows:

$$I_A = \frac{1}{4}\exp\left(-\frac{t_{\mathrm{mix}}}{T_1}\right)(1+\exp(-k_{\mathrm{obs}}t_{\mathrm{mix}}))M^0 \tag{S2}$$

and

$$I_B = \frac{1}{4}\exp\left(-\frac{t_{\mathrm{mix}}}{T_1}\right)(1-\exp(-k_{\mathrm{obs}}t_{\mathrm{mix}}))M^0 \tag{S3}$$

where t_{mix} is the exchange mixing time (d_8), T_1 is the relaxation time constant (assumed equal for A and B), k_{obs} is the exchange rate constant, where $k_{\mathrm{chem}} = 2k_{\mathrm{obs}}$,[S12] and M^0 is the initial magnetization. Taking the ratio of the intensities:

$$r = \frac{I_B}{I_A} \tag{S4}$$

$$r = \frac{1-\exp(-k_{obs}t_{\mathrm{mix}})}{1+\exp(-k_{obs}t_{\mathrm{mix}})} \tag{S5}$$

Fitting the experimental intensities (determined by deconvolution of the o_o and o_i resonances) to Equation S5 affords $k_{\mathrm{chem}} = 15\,\mathrm{s}^{-1}$ (Figure S25).

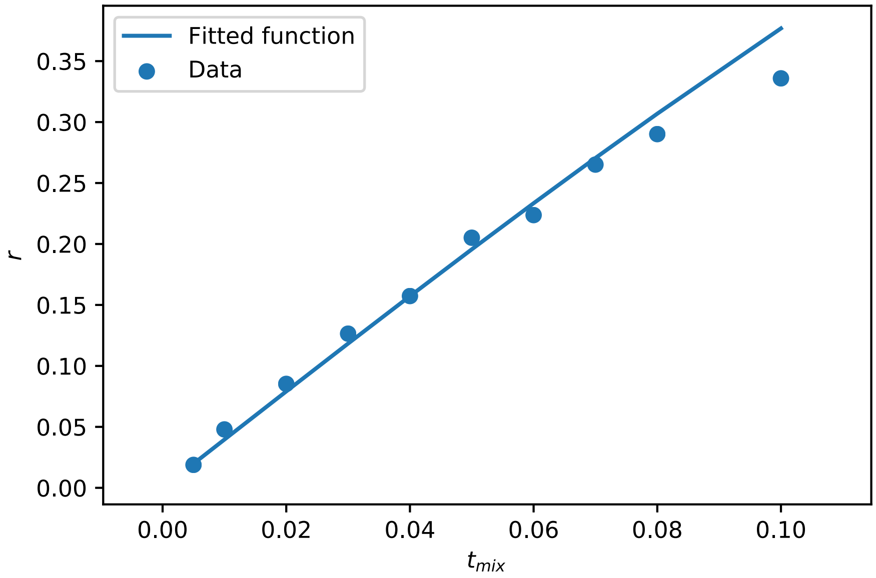

Figure S25: Experimental ratio of exchange intensities for the o_o and o_i resonances as a function of mixing time t_{mix}. The o_o resonance was selectively excited. ***c*-P6**$^{6-}$ (500 MHz, 228 K, d_8-THF). The line represents the fit of Equation S5, with $k_{\mathrm{chem}} = 15\,\mathrm{s}^{-1}$

.

We attempted EXSY experiments on ***c*-P6•T6**$^{6-}$, in which the inter-porphyrin rotation is restricted by the presence of the template. Any o_o to o_i EXSY would then arise from rotation of the *meso*-aryl group. EXSY measurements at 268 K revealed no reliable exchange peak between o_o and o_i, with any signal (either exchange or relayed nOe) only appearing at long mixing times. Accordingly, we conclude that the *meso*-aryl rotation does not contribute in the lower-temperature EXSY experiments on ***c*-P6**$^{6-}$ described above.

S3.6.3 Summary

In preference to using the error in the fit, we have applied an estimated error of 25% to the k_{chem} determined by EXSY for ***c*-P6**$^{6-}$. Propagation of the errors and calculation of the barrier height for rotation, using the Eyring equation, permits comparison of the barrier height in the neutral, 6−, 6+, and 12+ states (Table S4). The data for the 6+ and 12+ states has previously been reported.[S11]

Table S4: Summary of dynamic NMR data characterizing the φ_{A} rotation. Data for the 6+ and 12+ oxidation states are taken from the literature.[S11] T_c is estimated based on the frequency difference between the o_o and o_i resonances for ***c*-P6•T6** in the specified oxidation state, in a 500 MHz NMR spectrometer.

Oxidation state	T_{meas}	k_{chem} at T_{meas} (s^{-1})	$\Delta G^{\ddagger}_{T_{\mathrm{meas}}}$ (kJ/mol)	Estimated T_c
Neutral	202–203	577	38.0 ± 0.3	200–203
6−	228	15.1 ± 3.8	50.2 ± 0.5	295 ± 3
6+	213	3.5 ± 0.9	49.5 ± 0.4	282 ± 2
12+	213	172 ± 43	42.6 ± 0.4	234 ± 2

S4 Cartesian coordinates

The Cartesian coordinates listed below are also available in the native *XYZ* file format at DOI:10.6084/m9.figshare.9405308.

S4.1 *c*-P6 (B3LYP/6-31+G*)

```
234
cp6 neutral, D6h B3LYP/6-31+G*. E=-476542.38968499 au
C      2.8146740000   12.6650980000    1.2597510000
C      3.4838890000   12.6639140000    2.5397540000
C      2.5158220000   12.8197890000    3.4914240000
C      1.2525070000   12.8886550000    2.7975160000
N      1.4659610000   12.8091370000    1.4396140000
H      4.5515180000   12.5659510000    2.6823950000
H      2.6401940000   12.8692650000    4.5663410000
C      0.0000000000   12.9515540000    3.4128310000
C     -1.2525070000   12.8886550000    2.7975160000
C     -2.5158220000   12.8197890000    3.4914240000
N     -1.4659610000   12.8091370000    1.4396140000
C     -3.4838890000   12.6639140000    2.5397540000
H     -2.6401940000   12.8692650000    4.5663410000
C     -2.8146740000   12.6650980000    1.2597510000
H     -4.5515180000   12.5659510000    2.6823950000
C     -3.4592150000   12.5302700000    0.0000000000
C     -2.8146740000   12.6650980000   -1.2597510000
C     -3.4838890000   12.6639140000   -2.5397540000
N     -1.4659610000   12.8091370000   -1.4396140000
C     -2.5158220000   12.8197890000   -3.4914240000
H     -4.5515180000   12.5659510000   -2.6823950000
C     -1.2525070000   12.8886550000   -2.7975160000
H     -2.6401940000   12.8692650000   -4.5663410000
C      0.0000000000   12.9515540000   -3.4128310000
C      1.2525070000   12.8886550000   -2.7975160000
C      2.5158220000   12.8197890000   -3.4914240000
N      1.4659610000   12.8091370000   -1.4396140000
C      3.4838890000   12.6639140000   -2.5397540000
H      2.6401940000   12.8692650000   -4.5663410000
C      2.8146740000   12.6650980000   -1.2597510000
H      4.5515180000   12.5659510000   -2.6823950000
C      3.4592150000   12.5302700000    0.0000000000
Zn     0.0000000000   12.8159860000    0.0000000000
C      4.8230260000   12.1500270000    0.0000000000
C      5.9436010000   11.6497050000    0.0000000000
C     -4.8230260000   12.1500270000   -0.0000000000
C     -5.9436010000   11.6497050000   -0.0000000000
C    -11.2163750000    6.4757770000   -3.4128310000
C    -11.7881560000    5.3596240000   -2.7975160000
C    -10.5356490000    7.5290310000   -2.7975160000
C    -12.3601740000    4.2311280000   -3.4914240000
N    -11.8260190000    5.1350090000   -1.4396140000
C     -9.8443510000    8.5886600000   -3.4914240000
N    -10.3600570000    7.6741280000   -1.4396140000
C    -12.7092150000    3.3148210000   -2.5397540000
H    -12.4652070000    4.1481570000   -4.5663410000
C    -12.3756330000    3.8949700000   -1.2597510000
Zn   -11.0989700000    6.4079930000    0.0000000000
C     -9.2253270000    9.3490930000   -2.5397540000
H     -9.8250130000    8.7211080000   -4.5663410000
C     -9.5609590000    8.7701280000   -1.2597510000
H    -13.1581920000    2.3412450000   -2.6823950000
C    -12.5811400000    3.2693670000    0.0000000000
N    -10.3600570000    7.6741280000    1.4396140000
N    -11.8260190000    5.1350090000    1.4396140000
H     -8.6066730000   10.2247060000   -2.6823950000
C     -9.1219250000    9.2609030000   -0.0000000000
C    -12.3756330000    3.8949700000    1.2597510000
C    -12.9337450000    1.8981500000   -0.0000000000
C     -9.5609590000    8.7701280000    1.2597510000
C    -10.5356490000    7.5290310000    2.7975160000
C    -11.7881560000    5.3596240000    2.7975160000
C     -8.1107190000   10.2518760000    0.0000000000
C    -12.7092150000    3.3148210000    2.5397540000
C    -13.0607410000    0.6775430000   -0.0000000000
C     -9.2253270000    9.3490930000    2.5397540000
C     -9.8443510000    8.5886600000    3.4914240000
C    -11.2163750000    6.4757770000    3.4128310000
C    -12.3601740000    4.2311280000    3.4914240000
C     -7.1171400000   10.9721620000    0.0000000000
H    -13.1581920000    2.3412450000    2.6823950000
H     -8.6066730000   10.2247060000    2.6823950000
H     -9.8250130000    8.7211080000    4.5663410000
H    -12.4652070000    4.1481570000    4.5663410000
C     -7.1171400000  -10.9721620000   -0.0000000000
C     -5.9436010000  -11.6497050000    0.0000000000
C     -8.1107190000  -10.2518760000   -0.0000000000
C     -4.8230260000  -12.1500270000    0.0000000000
C     -9.1219250000   -9.2609030000    0.0000000000
C     -3.4592150000  -12.5302700000   -0.0000000000
C     -9.5609590000   -8.7701280000   -1.2597510000
C     -9.5609590000   -8.7701280000    1.2597510000
C     -2.8146740000  -12.6650980000   -1.2597510000
C     -2.8146740000  -12.6650980000    1.2597510000
C     -9.2253270000   -9.3490930000   -2.5397540000
N    -10.3600570000   -7.6741280000   -1.4396140000
C     -9.2253270000   -9.3490930000    2.5397540000
N    -10.3600570000   -7.6741280000    1.4396140000
N     -1.4659610000  -12.8091370000   -1.4396140000
C     -3.4838890000  -12.6639140000   -2.5397540000
N     -1.4659610000  -12.8091370000    1.4396140000
C     -3.4838890000  -12.6639140000    2.5397540000
C     -9.8443510000   -8.5886600000   -3.4914240000
H     -8.6066730000  -10.2247060000   -2.6823950000
C    -10.5356490000   -7.5290310000   -2.7975160000
Zn   -11.0989700000   -6.4079930000    0.0000000000
C     -9.8443510000   -8.5886600000    3.4914240000
H     -8.6066730000  -10.2247060000    2.6823950000
C    -10.5356490000   -7.5290310000    2.7975160000
C     -1.2525070000  -12.8886550000   -2.7975160000
Zn    -0.0000000000  -12.8159860000    0.0000000000
C     -2.5158220000  -12.8197890000   -3.4914240000
H     -4.5515180000  -12.5659510000   -2.6823950000
C     -1.2525070000  -12.8886550000    2.7975160000
C     -2.5158220000  -12.8197890000    3.4914240000
H     -4.5515180000  -12.5659510000    2.6823950000
H     -9.8250130000   -8.7211080000   -4.5663410000
C    -11.2163750000   -6.4757770000   -3.4128310000
N    -11.8260190000   -5.1350090000   -1.4396140000
N    -11.8260190000   -5.1350090000    1.4396140000
H     -9.8250130000   -8.7211080000    4.5663410000
C    -11.2163750000   -6.4757770000    3.4128310000
C     -0.0000000000  -12.9515540000   -3.4128310000
N      1.4659610000  -12.8091370000   -1.4396140000
N      1.4659610000  -12.8091370000    1.4396140000
H     -2.6401940000  -12.8692650000   -4.5663410000
C     -0.0000000000  -12.9515540000    3.4128310000
H     -2.6401940000  -12.8692650000    4.5663410000
C    -11.7881560000   -5.3596240000   -2.7975160000
C    -12.3756330000   -3.8949700000   -1.2597510000
C    -11.7881560000   -5.3596240000    2.7975160000
C    -12.3756330000   -3.8949700000    1.2597510000
C      1.2525070000  -12.8886550000   -2.7975160000
C      2.8146740000  -12.6650980000   -1.2597510000
C      2.8146740000  -12.6650980000    1.2597510000
C      1.2525070000  -12.8886550000    2.7975160000
C    -12.3601740000   -4.2311280000   -3.4914240000
C    -12.7092150000   -3.3148210000   -2.5397540000
C    -12.5811400000   -3.2693670000   -0.0000000000
C    -12.3601740000   -4.2311280000    3.4914240000
C    -12.7092150000   -3.3148210000    2.5397540000
C      2.5158220000  -12.8197890000   -3.4914240000
C      3.4838890000  -12.6639140000   -2.5397540000
C      3.4592150000  -12.5302700000    0.0000000000
C      3.4838890000  -12.6639140000    2.5397540000
C      2.5158220000  -12.8197890000    3.4914240000
H    -12.4652070000   -4.1481570000   -4.5663410000
H    -13.1581920000   -2.3412450000   -2.6823950000
C    -12.9337450000   -1.8981500000    0.0000000000
H    -12.4652070000   -4.1481570000    4.5663410000
H    -13.1581920000   -2.3412450000    2.6823950000
H      2.6401940000  -12.8692650000   -4.5663410000
H      4.5515180000  -12.5659510000   -2.6823950000
C      4.8230260000  -12.1500270000   -0.0000000000
H      4.5515180000  -12.5659510000    2.6823950000
H      2.6401940000  -12.8692650000    4.5663410000
C    -13.0607410000   -0.6775430000    0.0000000000
C      5.9436010000  -11.6497050000   -0.0000000000
C      7.1171400000  -10.9721620000    0.0000000000
C      8.1107190000  -10.2518760000    0.0000000000
C      9.1219250000   -9.2609030000   -0.0000000000
C      9.5609590000   -8.7701280000    1.2597510000
C      9.5609590000   -8.7701280000   -1.2597510000
C      9.2253270000   -9.3490930000    2.5397540000
N     10.3600570000   -7.6741280000    1.4396140000
N     10.3600570000   -7.6741280000   -1.4396140000
C      9.2253270000   -9.3490930000   -2.5397540000
C      9.8443510000   -8.5886600000    3.4914240000
H      8.6066730000  -10.2247060000    2.6823950000
C     10.5356490000   -7.5290310000    2.7975160000
Zn    11.0989700000   -6.4079930000    0.0000000000
C     10.5356490000   -7.5290310000   -2.7975160000
C      9.8443510000   -8.5886600000   -3.4914240000
```

```
H     8.6066730000  -10.2247060000  -2.6823950000
H     9.8250130000   -8.7211080000   4.5663410000
C    11.2163750000   -6.4757770000   3.4128310000
N    11.8260190000   -5.1350090000   1.4396140000
N    11.8260190000   -5.1350090000  -1.4396140000
C    11.2163750000   -6.4757770000  -3.4128310000
H     9.8250130000   -8.7211080000  -4.5663410000
C    11.7881560000   -5.3596240000   2.7975160000
C    12.3756330000   -3.8949700000   1.2597510000
C    12.3756330000   -3.8949700000  -1.2597510000
C    11.7881560000   -5.3596240000  -2.7975160000
C    12.3601740000   -4.2311280000   3.4914240000
C    12.7092150000   -3.3148210000   2.5397540000
C    12.5811400000   -3.2693670000   0.0000000000
C    12.7092150000   -3.3148210000  -2.5397540000
C    12.3601740000   -4.2311280000  -3.4914240000
H    12.4652070000   -4.1481570000   4.5663410000
H    13.1581920000   -2.3412450000   2.6823950000
C    12.9337450000   -1.8981500000  -0.0000000000
H    13.1581920000   -2.3412450000  -2.6823950000
H    12.4652070000   -4.1481570000  -4.5663410000
C    13.0607410000   -0.6775430000  -0.0000000000
C    13.0607410000    0.6775430000   0.0000000000
C    12.9337450000    1.8981500000   0.0000000000
C    12.5811400000    3.2693670000  -0.0000000000
C    12.3756330000    3.8949700000   1.2597510000
C    12.3756330000    3.8949700000  -1.2597510000
C    12.7092150000    3.3148210000   2.5397540000
N    11.8260190000    5.1350090000   1.4396140000
N    11.8260190000    5.1350090000  -1.4396140000
C    12.7092150000    3.3148210000  -2.5397540000
C    12.3601740000    4.2311280000   3.4914240000
H    13.1581920000    2.3412450000   2.6823950000
C    11.7881560000    5.3596240000   2.7975160000
Zn   11.0989700000    6.4079930000   0.0000000000
C    11.7881560000    5.3596240000  -2.7975160000
C    12.3601740000    4.2311280000  -3.4914240000
H    13.1581920000    2.3412450000  -2.6823950000
H    12.4652070000    4.1481570000   4.5663410000
C    11.2163750000    6.4757770000   3.4128310000
N    10.3600570000    7.6741280000   1.4396140000
N    10.3600570000    7.6741280000  -1.4396140000
C    11.2163750000    6.4757770000  -3.4128310000
H    12.4652070000    4.1481570000  -4.5663410000
C    10.5356490000    7.5290310000   2.7975160000
C     9.5609590000    8.7701280000   1.2597510000
C     9.5609590000    8.7701280000  -1.2597510000
C    10.5356490000    7.5290310000  -2.7975160000
C     9.8443510000    8.5886600000   3.4914240000
C     9.2253270000    9.3490930000   2.5397540000
C     9.1219250000    9.2609030000   0.0000000000
C     9.2253270000    9.3490930000  -2.5397540000
C     9.8443510000    8.5886600000  -3.4914240000
H     9.8250130000    8.7211080000   4.5663410000
H     8.6066730000   10.2247060000   2.6823950000
C     8.1107190000   10.2518760000  -0.0000000000
H     8.6066730000   10.2247060000  -2.6823950000
H     9.8250130000    8.7211080000  -4.5663410000
C     7.1171400000   10.9721620000  -0.0000000000
H   -11.2599900000    6.5009580000  -4.4987300000
H   -11.2599900000   -6.5009580000  -4.4987300000
H    -0.0000000000  -13.0019160000  -4.4987300000
H    11.2599900000   -6.5009580000  -4.4987300000
H    11.2599900000    6.5009580000  -4.4987300000
H     0.0000000000   13.0019160000  -4.4987300000
H    11.2599900000   -6.5009580000   4.4987300000
H    -0.0000000000  -13.0019160000   4.4987300000
H   -11.2599900000   -6.5009580000   4.4987300000
H   -11.2599900000    6.5009580000   4.4987300000
H     0.0000000000   13.0019160000   4.4987300000
H    11.2599900000    6.5009580000   4.4987300000
```

S4.2 *c*-P6^{4-} (B3LYP/6-31+G*)

```
234
cp6 tetraanion, C1 B3LYP/6-31+G*. E=-476546.84602277 au
 C        12.992838662940     -2.821959966239      1.268745754299
 C        13.021861760082     -3.485618304759      2.543632645412
 C        13.209403496054     -2.510896845110      3.495151107901
 C        13.272170560675     -1.256606800791      2.804569568631
 N        13.152610133984     -1.478213132994      1.439747449823
 H        12.912764796093     -4.552211028905      2.689717673160
 H        13.276503302841     -2.635821384403      4.570249515141
 C        13.349912290514      0.000270947907      3.408177884130
 C        13.272216527221      1.257165914925      2.804599053372
 C        13.209498255018      2.511442057897      3.495210022931
 N        13.152661883737      1.478808264171      1.439782331517
 C        13.021991435011      3.486192769534      2.543714413885
H        13.276604419202      2.636339096924      4.570311302089
C        12.992940050530      2.822565017744      1.268812463943
H        12.912934715463      4.552786187732      2.689824396859
C        12.810135551311      3.478786543719      0.000090283979
C        12.993066273162      2.822594897321     -1.268629293832
C        13.022246627321      3.486253251871     -2.543512329515
N        13.152802265511      1.478842000340     -1.439615335734
C        13.209847215299      2.511525015766     -3.495012553005
H        12.913207071023      4.552850449919     -2.689607619297
C        13.272493905343      1.257232191142     -2.804425320519
H        13.277061912005      2.636447815607     -4.570104050149
C        13.350249580031      0.000351547253     -3.408026248223
C        13.272448032903     -1.256540435178     -2.804455311670
C        13.209752341504     -2.510814494667     -3.495072451502
N        13.152751001990     -1.478178754282     -1.439650385539
C        13.022117178308     -3.485558497175     -2.543595458594
H        13.276960042382     -2.635713911117     -4.570167032119
C        12.992965305513     -2.821929821810     -1.268696137428
H        12.913036982143     -4.552148056623     -2.689716241447
C        12.810014492332     -3.478145796648      0.000007935987
Zn       13.140977661505      0.000315029869      0.000066092438
C        12.369056511231     -4.797118719049     -0.000029951189
C        11.838105327471     -5.919451619075     -0.000068324099
C        12.369226843506      4.797775846541      0.000084058134
C        11.838291437373      5.920115984105      0.000073244011
C         6.561798954392     11.113827240456     -3.408803745998
C         5.424854268611     11.638898475571     -2.800667902290
C         7.644886062079     10.463715539485     -2.799998843672
C         4.277998433084     12.167446555408     -3.492178064209
N         5.187780389761     11.662034348631     -1.436100769289
C         8.722717311376      9.820741809620     -3.493177712590
N         7.792633637664     10.272589013596     -1.439377684024
C         3.342817575417     12.477821711771     -2.541172457265
H         4.190734646548     12.266080359822     -4.568364552614
C         3.927635424672     12.162450943277     -1.259359860208
Zn        6.485912132589     10.959617375650      0.000065577225
C         9.506170456157      9.213375832494     -2.540194860756
H         8.856689626139      9.811402396889     -4.568990295576
C         8.913475437647      9.506649026735     -1.266019442246
H         2.349164002484     12.881242200209     -2.684287761840
C         3.281889385622     12.344812098175      0.000013897738
N         7.792541854610     10.272485471150      1.439542218668
N         5.187689315857     11.661931156729      1.436200259485
H        10.399381983999      8.620449968516     -2.685959651239
C         9.414360824717      9.047528222271      0.000089830346
C         3.927555112894     12.162359196483      1.259415430982
C         1.903510946763     12.653216501402     -0.000018681078
C         8.913397530755      9.506562410170      1.266199886720
C         7.644710678551     10.463518735037      2.800167438070
C         5.424676591796     11.638697177943      2.800780774666
C        10.402207410789      8.062451238931      0.000086857666
C         3.342655402394     12.477636668025      2.541213631728
C         0.677759546448     12.771797075984     -0.000047988379
C         9.506016445219      9.213205061924      2.540391294191
C         8.722502596465      9.820502544984      3.493367997753
C         6.561584049784     11.113584905652      3.408950674750
C         4.277776186376     12.167193975644      3.492256245295
C        11.134476354196      7.061608969300      0.000078718412
H         2.348992321008     12.881045829464      2.684294889938
H        10.399222178512      8.620273852622      2.686169860758
H         8.856409900019      9.811090833495      4.569188043376
H         4.190443875420     12.265749996218      4.568444314788
C       -11.134740488775      7.061217328009     -0.000175653820
C       -11.838478093443      5.919675073728     -0.000153872768
C       -10.402544916244      8.062112221219     -0.000182343621
C       -12.369384725688      4.797321715574     -0.000121020953
C        -9.414733212992      9.047223823613     -0.000150274710
C       -12.810265434931      3.478322399923     -0.000046817157
C        -8.913835224924      9.506369939046     -1.266245059751
C        -8.913816143038      9.506268363069      1.265973688846
C       -12.993198492417      2.822068955155     -1.268733191828
C       -12.993022401109      2.822152102190      1.268707925711
C        -9.506495663348      9.213091382447     -2.540435719048
N        -7.793012682192     10.272344622504     -1.439573407433
C        -9.506457821841      9.212887674931      2.540149485828
N        -7.792991105549     10.272229107457      1.439346958984
N       -13.152921581542      1.478306027810     -1.439654524788
C       -13.022401176543      3.485666869384     -2.543647604780
N       -13.152721543752      1.478400385117      1.439739708121
C       -13.022047777776      3.485833566938      2.543582865171
C        -8.723041186902      9.820489401173     -3.493396797791
H       -10.399687980719      8.620142431670     -2.686224233765
C        -7.645242883043     10.463488694233     -2.800189746554
Zn       -6.486350503867     10.959411857813     -0.000095278820
C        -8.722989639643      9.820209729382      3.493147779688
H       -10.399648124812      8.619927112999      2.685903477169
C        -7.645201515800     10.463264545893      2.799976437430
C       -13.272626720868      1.256630633216     -2.804452421355
Zn      -13.141015920757     -0.000158300322      0.000090223004
C       -13.210001618886      2.510892007802     -3.495099592289
H       -12.913374352461      4.552258203635     -2.689794758386
```

C	-13.272236346360	1.256814323951	2.804568802658
C	-13.209515322790	2.511121013050	3.495124971928
H	-12.913000943281	4.552434561840	2.689644836220
H	-8.856992662912	9.811158098355	-4.569212039153
C	-6.562162160004	11.113637503783	-3.408965969464
N	-5.188207057884	11.661875108421	-1.436227591557
N	-5.188186249735	11.661760930923	1.436074070487
H	-8.856925607318	9.810792659950	4.568964219172
C	-6.562112163870	11.113365106486	3.408788805883
C	-13.350362069994	-0.000279049830	-3.407995296148
N	-13.152779803271	-1.478716614462	-1.439557830318
N	-13.152578514943	-1.478622393933	1.439836801947
H	-13.277231827161	2.635763933587	-4.570195980442
C	-13.349887089526	-0.000055873792	3.408204888812
H	-13.276595825867	2.636063422770	4.570222530921
C	-5.425246896838	11.638738519559	-2.800800353485
C	-3.928081016112	12.162328301967	-1.259453303157
C	-5.425206010276	11.638515085203	2.800648470815
C	-3.928062838484	12.162228237317	1.259321311741
C	-13.272508469157	-1.257142160989	-2.804370069710
C	-12.992931420195	-2.822453417130	-1.268548377566
C	-12.992753973539	-2.822370438337	1.268893044941
C	-13.272117117150	-1.256958539496	2.804651193317
C	-4.278390574760	12.167324743623	-3.492280450795
C	-3.343242339630	12.477723562504	-2.541250527795
C	-3.282372552404	12.344706308248	-0.000063354574
C	-4.278339706944	12.167046376627	3.492153994945
C	-3.343205493650	12.477521350210	2.541135096394
C	-13.209768381722	-2.511442866043	-3.494934839164
C	-13.022075409070	-3.486137799234	-2.543418845560
C	-12.809933894607	-3.478606775867	0.000180990579
C	-13.021720000582	-3.485971280575	2.543811042946
C	-13.209280533297	-2.511214027011	3.495289349521
H	-4.191104554742	12.265968531208	-4.568464250530
H	-2.349598182088	12.881176265764	-2.684340118661
C	-1.904001028226	12.653143529355	-0.000060711282
H	-4.191037931769	12.265604318594	4.568347063763
H	-2.349559316689	12.880962912773	2.684242298535
H	-13.276988818539	-2.636391789322	-4.570022944673
H	-12.912949760609	-4.552728626012	-2.689496033862
C	-12.368924958918	-4.797562622391	0.000192636104
H	-12.912573955518	-4.552552542978	2.689942863556
H	-13.276351225938	-2.636092569255	4.570394988589
C	-0.678253252044	12.771771814405	-0.000058420524
C	-11.837914603920	-5.919867470594	0.000198868631
C	-11.134078673161	-7.061348790567	0.000198707535
C	-10.401844341005	-8.062216589701	0.000192619109
C	-9.414078482415	-9.047374060633	0.000170940096
C	-8.913105854599	-9.506436352282	1.266267550401
C	-8.913272336179	-9.506545324639	-1.265951935474
C	-9.505663342029	-9.213031493233	2.540476644402
N	-7.792301567194	-10.272442003240	1.439577371405
N	-7.792490802193	-10.272565693950	-1.439343440591
C	-9.505997486229	-9.213249730338	-2.540108203068
C	-8.722164841135	-9.820384557600	3.493430667479
H	-10.398821294685	-8.620034680747	2.686281343323
C	-7.644441524150	-10.463481723953	2.800198581672
Zn	-6.485772493053	-10.959681620715	0.000060446965
C	-7.644809692979	-10.463721818493	-2.799967717842
C	-8.722624339056	-9.820684380500	-3.493113192896
H	-10.399174536416	-8.620265301209	-2.685846257120
H	-8.856038804768	-9.810961088389	4.569254749680
C	-6.561342830778	-11.113624915876	3.408949362025
N	-5.187552098488	-11.662077612129	1.436156164214
N	-5.187740859130	-11.662200392417	-1.436145789543
C	-6.561790785543	-11.113916554295	-3.408805311717
H	-8.856639691356	-9.811352954292	-4.568920469198
C	-5.424492979852	-11.638821683682	2.800744289791
C	-3.927458952000	-12.162595517333	1.259331357997
C	-3.927624192868	-12.162702443570	-1.259443928291
C	-5.424860851936	-11.639060852531	-2.800704761205
C	-4.277607506370	-12.167397931730	3.492183653064
C	-3.342538317284	-12.477906536270	2.541111685942
C	-3.281848046537	-12.345101037548	-0.000090944591
C	-3.342871652391	-12.478122231389	-2.541274225627
C	-4.278065831077	-12.167695121157	-3.492249794572
H	-4.190247444320	-12.265958232987	4.568369068771
H	-2.348898537371	-12.881384067588	2.684162082342
C	-1.903489063291	-12.653593523855	-0.000168653845
H	-2.349250460592	-12.881611374608	-2.684420792385
H	-4.190846983603	-12.266346879073	-4.568438269637
C	-0.677743300113	-12.772242713094	-0.000225886827
C	0.678271353690	-12.772220877054	-0.000254359403
C	1.904018472288	-12.653591952176	-0.000264399727
C	3.282364338349	-12.345054906346	-0.000224751756
C	3.928000246736	-12.162528048884	1.259180254273
C	3.928091213930	-12.162610142968	-1.259595199996
C	3.343129067312	-12.477872532064	2.540975685898
N	5.188079879064	-11.661964603471	1.435972721048
N	5.188183555865	-11.662057775318	-1.436328791235
C	3.343313084554	-12.478038246233	-2.541412502401
C	4.278210760933	-12.167330028753	3.492023856461
H	2.349511419811	-12.881395826198	2.684050853205
C	5.425055576254	-11.638704321434	2.800554571458
Zn	6.486236896183	-10.959511660995	-0.000153882383
C	5.425257846830	-11.638885552483	-2.800895017518
C	4.278463428428	-12.167556733609	-3.492413059273
H	2.349705975294	-12.881571063238	-2.684533553028
H	4.190886153203	-12.265902337962	4.568211024658
C	6.561903447491	-11.113471347561	3.408731506209
N	7.792784316160	-10.272241093024	1.439330809770
N	7.792888130853	-10.272333642399	-1.439588645226
C	6.562149204332	-11.113690871270	-3.409023772269
H	4.191216717754	-12.266198662395	-4.568600159198
C	7.644963636555	-10.463288117295	2.799955106526
C	8.913558007686	-9.506195853163	1.265995612465
C	8.913648653579	-9.506276205285	-1.266222119507
C	7.645165102793	-10.463467677267	-2.800211312420
C	8.722680448033	-9.820153440523	3.493163169845
C	9.506133237531	-9.212769565249	2.540191826877
C	9.414480501620	-9.047108221810	-0.000109866364
C	9.506314567351	-9.212930239774	-2.540394675864
C	8.722930687369	-9.820375803548	-3.493383301489
H	8.856579480150	-9.810727234919	4.568984132191
H	10.399273268151	-8.619740864488	2.685976344427
C	10.402217819995	-8.061919788129	-0.000105362106
H	10.399464210031	-8.619909644818	-2.686153204039
H	8.856906575020	-9.811017850186	-4.569195284215
C	11.134385110342	-7.061003764771	-0.000093328987
H	6.587058123170	11.161290617864	-4.495763516881
H	-6.587398831536	11.161109120781	-4.495925868024
H	-13.421892035174	-0.000318049038	-4.494380123905
H	-6.587094146723	-11.161392222000	-4.495763524861
H	6.587416771056	-11.161145370641	-4.495983685981
H	13.421760361922	0.000363310835	-4.494412371581
H	-6.586503469471	-11.161007898386	4.495914944757
H	-13.421265654602	-0.000023713095	4.494599687239
H	-6.587333003570	11.160749984476	4.495752872291
H	6.586775459985	11.160971862212	4.495915351535
H	13.421314221850	0.000257003015	4.494571177515
H	6.587092704563	-11.160855883375	4.495696302820

S4.3 *c*-P6^{6-} (B3LYP/6-31+G*)

234
cp6 hexaanion, D6h B3LYP/6-31+G*. E= -476541.54276872 au

C	2.8299320000	12.7786770000	1.2645760000
C	3.4888000000	12.8051600000	2.5422290000
C	2.5118770000	12.9941790000	3.4926490000
C	1.2584040000	13.0606820000	2.8004400000
N	1.4828830000	12.9423810000	1.4358730000
H	4.5545130000	12.6876450000	2.6898030000
H	2.6344950000	13.0546600000	4.5689710000
C	0.0000000000	13.1389640000	3.4035570000
C	-1.2584040000	13.0606820000	2.8004400000
C	-2.5118770000	12.9941790000	3.4926490000
N	-1.4828830000	12.9423810000	1.4358730000
C	-3.4888000000	12.8051600000	2.5422290000
H	-2.6344950000	13.0546600000	4.5689710000
C	-2.8299320000	12.7786770000	1.2645760000
H	-4.5545130000	12.6876450000	2.6898030000
C	-3.4911500000	12.5979530000	0.0000000000
C	-2.8299320000	12.7786770000	-1.2645760000
C	-3.4888000000	12.8051600000	-2.5422290000
N	-1.4828830000	12.9423810000	-1.4358730000
C	-2.5118770000	12.9941790000	-3.4926490000
H	-4.5545130000	12.6876450000	-2.6898030000
C	-1.2584040000	13.0606820000	-2.8004400000
H	-2.6344950000	13.0546600000	-4.5689710000
C	0.0000000000	13.1389640000	-3.4035570000
C	1.2584040000	13.0606820000	-2.8004400000
C	2.5118770000	12.9941790000	-3.4926490000
N	1.4828830000	12.9423810000	-1.4358730000
C	3.4888000000	12.8051600000	-2.5422290000
H	2.6344950000	13.0546600000	-4.5689710000
C	2.8299320000	12.7786770000	-1.2645760000
H	4.5545130000	12.6876450000	-2.6898030000
C	3.4911500000	12.5979530000	0.0000000000
Zn	0.0000000000	12.9252090000	0.0000000000
C	4.8235040000	12.1728140000	0.0000000000
C	5.9500050000	11.6532100000	0.0000000000
C	-4.8235040000	12.1728140000	-0.0000000000
C	-5.9500050000	11.6532100000	-0.0000000000
C	-11.3786770000	6.5694820000	-3.4035570000
C	-11.9400850000	5.4405310000	-2.8004400000
C	-10.6816800000	7.6201510000	-2.8004400000
C	-12.5092280000	4.3217410000	-3.4926490000
N	-11.9498720000	5.1869770000	-1.4358730000
C	-9.9973510000	8.6724390000	-3.4926490000

N	-10.4669900000	7.7554040000	-1.4358730000
C	-12.8339940000	3.3811900000	-2.5422290000
H	-12.6229150000	4.2457910000	-4.5689710000
C	-12.4816250000	3.9385450000	-1.2645760000
Zn	-11.1935590000	6.4626040000	0.0000000000
C	-9.3451940000	9.4239700000	-2.5422290000
H	-9.9884200000	8.8088690000	-4.5689710000
C	-9.6516930000	8.8401320000	-1.2645760000
H	-13.2650790000	2.3994990000	-2.6898030000
C	-12.6557220000	3.2755520000	0.0000000000
N	-10.4669900000	7.7554040000	1.4358730000
N	-11.9498720000	5.1869770000	1.4358730000
H	-8.7105660000	10.2881460000	-2.6898030000
C	-9.1645720000	9.3224010000	-0.0000000000
C	-12.4816250000	3.9385450000	1.2645760000
C	-12.9537190000	1.9091300000	-0.0000000000
C	-9.6516930000	8.8401320000	1.2645760000
C	-10.6816800000	7.6201510000	2.8004400000
C	-11.9400850000	5.4405310000	2.8004400000
C	-8.1302150000	10.2636840000	0.0000000000
C	-12.8339940000	3.3811900000	2.5422290000
C	-13.0669790000	0.6737500000	-0.0000000000
C	-9.3451940000	9.4239700000	2.5422290000
C	-9.9973510000	8.6724390000	3.4926490000
C	-11.3786770000	6.5694820000	3.4035570000
C	-12.5092280000	4.3217410000	3.4926490000
C	-7.1169740000	10.9794600000	0.0000000000
H	-13.2650790000	2.3994990000	2.6898030000
H	-8.7105660000	10.2881460000	2.6898030000
H	-9.9884200000	8.8088690000	4.5689710000
H	-12.6229150000	4.2457910000	4.5689710000
C	-7.1169740000	-10.9794600000	-0.0000000000
C	-5.9500050000	-11.6532100000	0.0000000000
C	-8.1302150000	-10.2636840000	-0.0000000000
C	-4.8235040000	-12.1728140000	0.0000000000
C	-9.1645720000	-9.3224010000	0.0000000000
C	-3.4911500000	-12.5979530000	-0.0000000000
C	-9.6516930000	-8.8401320000	-1.2645760000
C	-9.6516930000	-8.8401320000	1.2645760000
C	-2.8299320000	-12.7786770000	-1.2645760000
C	-2.8299320000	-12.7786770000	1.2645760000
C	-9.3451940000	-9.4239700000	-2.5422290000
N	-10.4669900000	-7.7554040000	-1.4358730000
C	-9.3451940000	-9.4239700000	2.5422290000
N	-10.4669900000	-7.7554040000	1.4358730000
N	-1.4828830000	-12.9423810000	-1.4358730000
C	-3.4888000000	-12.8051600000	-2.5422290000
N	-1.4828830000	-12.9423810000	1.4358730000
C	-3.4888000000	-12.8051600000	2.5422290000
C	-9.9973510000	-8.6724390000	-3.4926490000
H	-8.7105660000	-10.2881460000	-2.6898030000
C	-10.6816800000	-7.6201510000	-2.8004400000
Zn	-11.1935590000	-6.4626040000	0.0000000000
C	-9.9973510000	-8.6724390000	3.4926490000
H	-8.7105660000	-10.2881460000	2.6898030000
C	-10.6816800000	-7.6201510000	2.8004400000
C	-1.2584040000	-13.0606820000	-2.8004400000
Zn	-0.0000000000	-12.9252090000	0.0000000000
C	-2.5118770000	-12.9941790000	-3.4926490000
H	-4.5545130000	-12.6876450000	-2.6898030000
C	-1.2584040000	-13.0606820000	2.8004400000
C	-2.5118770000	-12.9941790000	3.4926490000
H	-4.5545130000	-12.6876450000	2.6898030000
H	-9.9884200000	-8.8088690000	-4.5689710000
C	-11.3786770000	-6.5694820000	-3.4035570000
N	-11.9498720000	-5.1869770000	-1.4358730000
N	-11.9498720000	-5.1869770000	1.4358730000
H	-9.9884200000	-8.8088690000	4.5689710000
C	-11.3786770000	-6.5694820000	3.4035570000
C	-0.0000000000	-13.1389640000	-3.4035570000
N	1.4828830000	-12.9423810000	-1.4358730000
N	1.4828830000	-12.9423810000	1.4358730000
H	-2.6344950000	-13.0546600000	-4.5689710000
C	-0.0000000000	-13.1389640000	3.4035570000
H	-2.6344950000	-13.0546600000	4.5689710000
C	-11.9400850000	-5.4405310000	-2.8004400000
C	-12.4816250000	-3.9385450000	-1.2645760000
C	-11.9400850000	-5.4405310000	2.8004400000
C	-12.4816250000	-3.9385450000	1.2645760000
C	1.2584040000	-13.0606820000	-2.8004400000
C	2.8299320000	-12.7786770000	-1.2645760000
C	2.8299320000	-12.7786770000	1.2645760000
C	1.2584040000	-13.0606820000	2.8004400000
C	-12.5092280000	-4.3217410000	-3.4926490000
C	-12.8339940000	-3.3811900000	-2.5422290000
C	-12.6557220000	-3.2755520000	-0.0000000000
C	-12.5092280000	-4.3217410000	3.4926490000
C	-12.8339940000	-3.3811900000	2.5422290000
C	2.5118770000	-12.9941790000	-3.4926490000
C	3.4888000000	-12.8051600000	-2.5422290000
C	3.4911500000	-12.5979530000	0.0000000000
C	3.4888000000	-12.8051600000	2.5422290000
C	2.5118770000	-12.9941790000	3.4926490000
H	-12.6229150000	-4.2457910000	-4.5689710000
H	-13.2650790000	-2.3994990000	-2.6898030000
C	-12.9537190000	-1.9091300000	0.0000000000
H	-12.6229150000	-4.2457910000	4.5689710000
H	-13.2650790000	-2.3994990000	2.6898030000
H	2.6344950000	-13.0546600000	-4.5689710000
H	4.5545130000	-12.6876450000	-2.6898030000
C	4.8235040000	-12.1728140000	-0.0000000000
H	4.5545130000	-12.6876450000	2.6898030000
H	2.6344950000	-13.0546600000	4.5689710000
C	-13.0669790000	-0.6737500000	0.0000000000
C	5.9500050000	-11.6532100000	-0.0000000000
C	7.1169740000	-10.9794600000	0.0000000000
C	8.1302150000	-10.2636840000	0.0000000000
C	9.1645720000	-9.3224010000	-0.0000000000
C	9.6516930000	-8.8401320000	1.2645760000
C	9.6516930000	-8.8401320000	-1.2645760000
C	9.3451940000	-9.4239700000	2.5422290000
N	10.4669900000	-7.7554040000	1.4358730000
N	10.4669900000	-7.7554040000	-1.4358730000
C	9.3451940000	-9.4239700000	-2.5422290000
C	9.9973510000	-8.6724390000	3.4926490000
H	8.7105660000	-10.2881460000	2.6898030000
C	10.6816800000	-7.6201510000	2.8004400000
Zn	11.1935590000	-6.4626040000	0.0000000000
C	10.6816800000	-7.6201510000	-2.8004400000
C	9.9973510000	-8.6724390000	-3.4926490000
H	8.7105660000	-10.2881460000	-2.6898030000
H	9.9884200000	-8.8088690000	4.5689710000
C	11.3786770000	-6.5694820000	3.4035570000
N	11.9498720000	-5.1869770000	1.4358730000
N	11.9498720000	-5.1869770000	-1.4358730000
C	11.3786770000	-6.5694820000	-3.4035570000
H	9.9884200000	-8.8088690000	-4.5689710000
C	11.9400850000	-5.4405310000	2.8004400000
C	12.4816250000	-3.9385450000	1.2645760000
C	12.4816250000	-3.9385450000	-1.2645760000
C	11.9400850000	-5.4405310000	-2.8004400000
C	12.5092280000	-4.3217410000	3.4926490000
C	12.8339940000	-3.3811900000	2.5422290000
C	12.6557220000	-3.2755520000	0.0000000000
C	12.8339940000	-3.3811900000	-2.5422290000
C	12.5092280000	-4.3217410000	-3.4926490000
H	12.6229150000	-4.2457910000	4.5689710000
H	13.2650790000	-2.3994990000	2.6898030000
C	12.9537190000	-1.9091300000	-0.0000000000
H	13.2650790000	-2.3994990000	-2.6898030000
H	12.6229150000	-4.2457910000	-4.5689710000
C	13.0669790000	-0.6737500000	-0.0000000000
C	13.0669790000	0.6737500000	0.0000000000
C	12.9537190000	1.9091300000	0.0000000000
C	12.6557220000	3.2755520000	-0.0000000000
C	12.4816250000	3.9385450000	1.2645760000
C	12.4816250000	3.9385450000	-1.2645760000
C	12.8339940000	3.3811900000	2.5422290000
N	11.9498720000	5.1869770000	1.4358730000
N	11.9498720000	5.1869770000	-1.4358730000
C	12.8339940000	3.3811900000	-2.5422290000
C	12.5092280000	4.3217410000	3.4926490000
H	13.2650790000	2.3994990000	2.6898030000
C	11.9400850000	5.4405310000	2.8004400000
Zn	11.1935590000	6.4626040000	0.0000000000
C	11.9400850000	5.4405310000	-2.8004400000
C	12.5092280000	4.3217410000	-3.4926490000
H	13.2650790000	2.3994990000	-2.6898030000
H	12.6229150000	4.2457910000	4.5689710000
C	11.3786770000	6.5694820000	3.4035570000
N	10.4669900000	7.7554040000	1.4358730000
N	10.4669900000	7.7554040000	-1.4358730000
C	11.3786770000	6.5694820000	-3.4035570000
H	12.6229150000	4.2457910000	-4.5689710000
C	10.6816800000	7.6201510000	2.8004400000
C	9.6516930000	8.8401320000	1.2645760000
C	9.6516930000	8.8401320000	-1.2645760000
C	10.6816800000	7.6201510000	-2.8004400000
C	9.9973510000	8.6724390000	3.4926490000
C	9.3451940000	9.4239700000	2.5422290000
C	9.1645720000	9.3224010000	0.0000000000
C	9.3451940000	9.4239700000	-2.5422290000
C	9.9973510000	8.6724390000	-3.4926490000
H	9.9884200000	8.8088690000	4.5689710000
H	8.7105660000	10.2881460000	2.6898030000
C	8.1302150000	10.2636840000	-0.0000000000
H	8.7105660000	10.2881460000	-2.6898030000
H	9.9884200000	8.8088690000	-4.5689710000
C	7.1169740000	10.9794600000	-0.0000000000
H	-11.4362580000	6.6027270000	-4.4907680000
H	-11.4362580000	-6.6027270000	-4.4907680000
H	-0.0000000000	-13.2054530000	-4.4907680000

H	11.4362580000	-6.6027270000	-4.4907680000
H	11.4362580000	6.6027270000	-4.4907680000
H	0.0000000000	13.2054530000	-4.4907680000
H	11.4362580000	-6.6027270000	4.4907680000
H	-0.0000000000	-13.2054530000	4.4907680000
H	-11.4362580000	-6.6027270000	4.4907680000
H	-11.4362580000	6.6027270000	4.4907680000
H	0.0000000000	13.2054530000	4.4907680000
H	11.4362580000	6.6027270000	4.4907680000

References

[S1] A. D. Becke, *J. Chem. Phys.* **1993**, *98*, 5648–5652.

[S2] W. J. Hehre, R. Ditchfield, J. A. Pople, *J. Chem. Phys.* **1972**, *56*, 2257–2261.

[S3] V. A. Rassolov, J. A. Pople, M. A. Ratner, T. L. Windus, *J. Chem. Phys.* **1998**, *109*, 1223–1229.

[S4] R. Ditchfield, W. J. Hehre, J. A. Pople, *J. Chem. Phys.* **1971**, *54*, 724–728.

[S5] P. C. Hariharan, J. A. Pople, *Theor. Chim. Acta* **1973**, *28*, 213–222.

[S6] T. Clark, J. Chandrasekhar, G. W. Spitznagel, P. v. R. Schleyer, *Journal of Computational Chemistry* **1983**, *4*, 294–301.

[S7] J.-D. Chai, M. Head-Gordon, *Phys. Chem. Chem. Phys.* **2008**, *10*, 6615–6620.

[S8] Y. Zhao, D. G. Truhlar, *Theor. Chem. Acc.* **2008**, *120*, 215–241.

[S9] M. J. Frisch, G. W. Trucks, H. B. Schlegel, G. E. Scuseria, M. A. Robb, J. R. Cheeseman, G. Scalmani, V. Barone, G. A. Petersson, H. Nakatsuji, X. Li, M. Caricato, A. V. Marenich, J. Bloino, B. G. Janesko, R. Gomperts, B. Mennucci, H. P. Hratchian, J. V. Ortiz, A. F. Izmaylov, J. L. Sonnenberg, D. Williams-Young, F. Ding, F. Lipparini, F. Egidi, J. Goings, B. Peng, A. Petrone, T. Henderson, D. Ranasinghe, V. G. Zakrzewski, J. Gao, N. Rega, G. Zheng, W. Liang, M. Hada, M. Ehara, K. Toyota, R. Fukuda, J. Hasegawa, M. Ishida, T. Nakajima, Y. Honda, O. Kitao, H. Nakai, T. Vreven, K. Throssell, J. A. Montgomery Jr., J. E. Peralta, F. Ogliaro, M. J. Bearpark, J. J. Heyd, E. N. Brothers, K. N. Kudin, V. N. Staroverov, T. A. Keith, R. Kobayashi, J. Normand, K. Raghavachari, A. P. Rendell, J. C. Burant, S. S. Iyengar, J. Tomasi, M. Cossi, J. M. Millam, M. Klene, C. Adamo, R. Cammi, J. W. Ochterski, R. L. Martin, K. Morokuma, O. Farkas, J. B. Foresman, D. J. Fox, Gaussian 16 Revision A.03, Gaussian Inc. Wallingford CT, **2016**.

[S10] C. E. Tait, P. Neuhaus, M. D. Peeks, H. L. Anderson, C. R. Timmel, *J. Am. Chem. Soc.* **2015**, *137*, 8284–8293.

[S11] M. D. Peeks, T. D. W. Claridge, H. L. Anderson, *Nature* **2017**, *541*, 200–203.

[S12] M. L. H. Green, L. L. Wong, A. Sella, *Organometallics* **1992**, *11*, 2660–2668.
